# VEGF-A in serum protects against memory impairment in APP/PS1 transgenic mice by blocking neutrophil infiltration

**DOI:** 10.1038/s41380-023-02097-w

**Published:** 2023-06-06

**Authors:** Fangfang Qi, Zejie Zuo, Kaishun Hu, Rui Wang, Tong Wu, Hao Liu, Jiaoling Tang, Qingbo Wang, Yufeng Xie, Liren Tan, Yunjie Yang, Xiaoran Zhang, Jiaying Zheng, Jie Xu, Zhibin Yao, Shengwen Wang, Long-Jun Wu, Kaihua Guo

**Affiliations:** 1 Department of Anatomy and Physiology, Guangdong Province Key Laboratory of Brain Function and Disease, Advanced Medical Technology Center, The First Affiliated Hospital, Zhongshan School of Medicine, Sun Yat-sen University, Guangzhou, 510080 China; 2 Editorial Department of Journal of Sun Yat-sen University, Guangzhou, 510080 China; 3 Department of Rehabilitation Medicine, the Third Affiliated Hospital, Sun Yat-sen University, Guangzhou, 510630 China; 4 Guangdong Provincial Key Laboratory of Malignant Tumor Epigenetics and Gene Regulation, Guangdong-Hong Kong Joint Laboratory for RNA Medicine, Sun Yat-sen Memorial Hospital, Sun Yat-sen University, Guangzhou, 510120 China; 5 Five-year Programs of Clinical Medicine in the 2017 grade, School of Medicine, Sun Yat-sen University, Shenzhen, 528406 China; 6 Department of Neurology, Sun Yat-sen Memorial Hospital, Sun Yat-sen University, Guangzhou, 510120 China; 7 Center for Stem Cell Biology and Tissue Engineering, Key Laboratory for Stem Cells and Tissue Engineering, Ministry of Education, Sun Yat-sen University, Guangzhou, 510080 China; 8 Department of Neurology, Mayo Clinic, Rochester, MN 55905 USA; 9 Department of Neurosurgery, Sun Yat-sen Memorial Hospital, Sun Yat-sen University, Guangzhou, 510120 China; 10 Present Address: Department of Neurology, Mayo Clinic, Rochester, MN 55905 USA

**Keywords:** Neuroscience, Molecular biology, Cell biology

## Abstract

Activation of innate immunity in the brain is a prominent feature of Alzheimer’s disease (AD). The present study investigated the regulation of innate immunity by wild-type serum injection in a transgenic AD mouse model. We found that treatment with wild-type mouse serum significantly reduced the number of neutrophils and microglial reactivity in the brains of APP/PS1 mice. Mimicking this effect, neutrophil depletion via Ly6G neutralizing antibodies resulted in improvements in AD brain functions. Serum proteomic analysis identified vascular endothelial growth factor-A (VEGF-A) and chemokine (C-X-C motif) ligand 1 (CXCL1) as factors enriched in serum samples, which are crucial for neutrophil migration and chemotaxis, leukocyte migration, and cell chemotaxis. Exogenous VEGF-A reversed amyloid β (Aβ)-induced decreases in cyclin-dependent kinase 5 (Cdk5) and increases in CXCL1 in vitro and blocked neutrophil infiltration into the AD brain. Endothelial Cdk5 overexpression conferred an inhibitory effect on CXCL1 and neutrophil infiltration, thereby restoring memory abilities in APP/PS1 mice. Our findings uncover a previously unknown link between blood-derived VEGF signaling and neutrophil infiltration and support targeting endothelial Cdk5 signaling as a potential therapeutic strategy for AD.

## Introduction

Alzheimer’s disease (AD) is the prevalent type of dementia that primarily affects elderly patients. Amyloid beta (Aβ) and neurofibrillary tangles (NFTs) in the brain are the main pathological features of AD. Increasing evidence suggests that circulatory factors, including immune cells and related cytokines, may be associated with pathological changes in AD mouse models [1–5]. Neutrophils, as the most abundant innate leukocytes in the peripheral blood, have been found to be increased in the blood of AD patients with dementia [6], in the brain parenchyma of AD patients [7, 8], and in several AD mouse models [4, 9–11]. In addition, systemic factors in the blood of young mice can rejuvenate neurogenesis, synaptic plasticity, and cognitive functions in aging mice [12, 13] and vice versa [14], indicating that systemic factors play an important role in modulating host behavior, immunity, and brain functions [15–17]. Thus, these studies suggest that innate immunity, including neutrophils, may play a crucial role in the development of AD [4, 6–8, 18]. However, little is known about how this mechanism may be related to therapies using young blood and other therapeutic strategies involving circulating factors. Moreover, the key systemic factors underlying the homeostasis of innate immunity in the AD brain remain to be determined.

As a blood-borne factor, vascular endothelial growth factor-A (VEGF-A) signaling has been shown to be involved in halting AD-related neurodegeneration and cognitive decline [19–21]. Insufficient VEGF signaling has been found to impact multiorgan aging, including brain cognition in mice, whereas enhanced VEGF signaling can prevent age-associated capillary loss in the brain and extend lifespan [22]. VEGF-A can also increase hippocampal neurogenesis in young mice [23, 24], and signal to the hippocampal endothelial cells, which are essential sensors of age-related circulatory factors [25]. VEGF has been found to prevent Aβ-induced AMPA-receptor loss and rescue synaptic dysfunction through direct action on synapses [26].

In this study, we revealed that the numbers of inflammatory neutrophils were decreased in the periphery and the brain in APP/PS1 mice after wild-type serum injection. We further sought to uncover the role of endothelial VEGF-A/cyclin-dependent kinase 5 (Cdk5)/ chemokine (C-X-C motif) ligand 1 (CXCL1) signaling in the process of neutrophil infiltration into the brain of APP/PS1 mice. Our results demonstrate that targeting neutrophils and/or trafficking molecule signaling to rebalance the immune microenvironment in the brain is a promising strategy for AD therapy.

## Materials and methods

### Animals

Eight-month-old male APP/PS1 (APPswePSEN1dE9) transgenic mice on a C57BL/6 background, age-matched wild-type (WT) mice, and 4- to 6-week-old male C57BL/6 mice were obtained from the Nanjing Biomedical Research Institute of Nanjing University (Nanjing, China). The promoters of both transgenes in the APP/PS1 transgenic mice coexpressing the human KM670/671L-mutated APP gene and M146L-mutated PSEN1 gene are controlled by the mouse prion protein read-through transcript (Prn). *Cx3cr1*-GFP transgenic mice (B6.129P-*Cx3cr1*^tm1Litt^/J) were purchased from the Jackson Laboratory (Stock #: 005582). The animals were anesthetized using an intraperitoneal (i.p.) injection of 250 mg/kg tribromoethanol (T48402, Sigma-Aldrich, St. Louis, MO, USA) and 2.5% tert-amyl alcohol (240486, Sigma-Aldrich, St. Louis, MO, USA) filtered with a 0.22-µm filter (Millipore, MA, USA), blood was collected, and the brain was perfused with cold saline and/or 4% PFA before harvesting. All animals were housed under temperature- and humidity-controlled conditions and maintained in a 12-h light/dark cycle environment. All experimental protocols complied with the regulations of the Institutional Animal Care and Use Committee of Sun Yat-sen University. Animals were excluded from the experiments when showing an obvious state of disease-like behavior, such as being motionless in the OFT and MWM tests or dying before or during the experiments.

### Serum collection and administration

Mouse serum was collected from young male C57BL/6 mice (*n* = 150; 4–6 weeks old) after retro-orbital bleeding. Serum was prepared from the whole blood after incubation for 1 h at room temperature and at 4 °C overnight and centrifugation at 4000 × *g*. Pooled mouse serum was filtered through a 0.22-µm pore size sterile syringe filter (Millipore), dialyzed using a Slide-A-Lyzer™ cassette (Thermo Scientific), and then stored at −80 °C. Eight-month-old male APP/PS1 mice (*n* = 20) and age-matched WT littermates (*n* = 20) were systemically treated with 9 injections of serum (200 μL/injection) or an equal amount of sterile PBS in the tail vein every 3 days. All mice (~9 months old) were sacrificed 48 h after the fear conditioning test; *n* = 7–10 for the behavioral tests and *n* = 4–15 for histological and biochemical assays [27]. The sample size was chosen based on similar previous studies in AD [3, 4, 10]. All samples were randomly assigned to groups with approximately balanced sample sizes.

### Fear conditioning

Fear conditioning was conducted in a chamber with internal dimensions of 20 cm width × 20 cm depth × 30 cm height (Coulbourn Instruments, USA). Male mice were individually placed in the test chamber. After a baseline time (192 s), the animals received three tone-shock pairings. The conditioned stimulus (CS) was a pure tone (20 s duration, 4000 Hz, 60 dB), which was immediately followed by a continuously scrambled electric shock delivered on the grid floor (unconditioned stimulus, US: 2 s, 0.5 mA). Each tone-shock pairing was followed by 64 s of time, and mice remained in context A for another 64 s after the third shock. Twenty-four hours after conditioning, the mice were placed back in the test chamber for 320 s and were scored for freezing time (contextual conditioning). Subsequently, the animals were moved to a new chamber with unscented black plastic internal walls and bedding on the floor and were scored for freezing during a 192-s baseline period followed by 320-s exposure to a tone identical to the conditioned stimulus (cued conditioning). The detailed protocol has been described previously [28].

### Morris water maze (MWM)

MWM test was conducted using male mice, as previously described with slight modification [29]. Briefly, during the acquisition phase, mice were given one training trial per day to locate a hidden platform positioned 1.0 cm below the water surface in a pool, using the distinct visual cues placed on the inner wall of the pool. The maximum time given for each trial was 60 s. One mouse failed to reach the platform within 60 s and was manually guided to the platform and remained for 10 s before it was returned to the cage. The intertrial interval for each mouse was 10 min. During the probe phase, the platform was removed, and mice were given a single trial lasting 60 s without available escape. Swimming trajectories were recorded with the TopScan^TM^ 2.0 (Clever Sys., Inc., Reston, VA, USA) Video tracking system. All behavioral tests were performed between 10:00 and 17:00 in the light-off phase.

### Tissue preparation and brain protein extraction

Under deep anesthesia, mice were transcardially perfused with cold sterile saline, and the brain was immediately removed. One hemisphere was frozen in liquid nitrogen, and the other hemisphere was immersion-fixed in 4% paraformaldehyde at 4 °C for 24 h and equilibrated in 15% sucrose in 0.1 M phosphate buffer (PB) followed by 30% sucrose. Then, 40-μm-thick frozen sections of the hippocampus were collected using a Leica SM2000R sliding microtome (Leica Microsystems, Richmond Hill, Ontario, Canada) and stored at 4 °C until immunofluorescence staining was performed. The brain slices were selected at a continuous equidistance of five coronal slices spaced 240 μm apart. The frozen hemispheres were homogenized and extracted at 4 °C in RIPA (radioimmunoprecipitation assay) buffer (50 mM Tris-HCl, pH 7.4, 150 mM NaCl, 1% Triton X-100, 1% sodium deoxycholate, and 0.1% SDS) containing 1% phosphatase and 1% protease inhibitors (Sigma-Aldrich, MO, USA) using a TissueRuptor homogenizer (Qiagen, DUS, Germany); the homogenate was centrifuged for 30 min at 12,000 × *g* (Eppendorf, HAM, Germany), and the supernatant was collected as the RIPA-soluble fraction. The pellet was re-extracted in 2% SDS and 50 mM Tris-HCl, pH 7.4. The supernatant was collected as the SDS-soluble fraction.

### Antibodies and reagents for immunofluorescence

The following primary antibodies were used: mouse anti-Aβ_1-42_ (1:1,000; A5213, Sigma-Aldrich), rat anti-CD68 (1:400; MCA1957, Bio-Rad), rabbit anti-Iba-1 (1:1,000; Wako Chemical), mouse anti-glutamate receptor 1 (1:400; ab183797, Abcam), mouse anti-synaptophysin (1:200; S5768, Sigma-Aldrich), rat anti-mouse Ly6G (1:200; BP0075, Bioxcell), mouse anti-myeloperoxidase (MPO, 1:1,000; AF3667-SP, R&D), mouse anti neutrophil elastase (NE, 1:1 000; MAB4517-SP, R&D), rat anti-Ki67 (1:500; SolA15, eBioscience), rabbit anti-Claudin5 polyclonal antibody (1:400; YT0953, Immunoway) mouse anti-pCdk5 (1:400; C-7, Santa Cruz) and mouse anti-Cdk5 (1:400; DC 17, Santa Cruz). The following secondary antibodies were used: Alexa Fluor 647 donkey anti-mouse, Alexa Fluor 594 donkey anti-rat, Alexa Fluor 488 donkey anti-goat, Alexa Fluor 555 goat anti-rabbit, and Alexa Fluor 488 goat anti-rabbit (1:400, Invitrogen). PE-conjugated anti-CD31 (1:100; 553373, B&D), FITC-conjugated anti-CXCL1 (1:100; IC4532G, B&D) and DyLight 649-labeled lectin (1:200; L32472, Invitrogen^TM^) antibodies were used in some immunofluorescence experiments. The detailed immunofluorescence protocols have been described previously [30].

### Confocal microscopy and three-dimensional reconstruction

An LSM 780 or 800 confocal laser scanning microscope (Carl Zeiss Microscopy) was used to capture representative images of the sections using the same parameters to avoid potential technical artefacts. ImageJ software (NIH) was used for the mean intensity of fluorescence signal quantification in the region of interest in each image. For analysis of colocalization in Supplementary Fig. 4, fluorescence intensity profile distribution analysis of Cdk5 and pCdk5 with CXCL1 using ZEN 2.3 (Carl Zeiss Microscopy). In detailed description, open a confocal fluorescence microscopy using ZEN 2.3 (blue edition) and select “Processing”, then select “Profile definition”, the fluorescence signal distribution could be showed in graphics across the arrow. The fluorescence intensity data list could be found in the “Profile Table”. For 3D reconstruction, the original confocal Z-stack images were obtained using a 63× oil immersion lens with digital Zoom 2.0 by LSM 780 microscope. Then, Imaris software (Bitplane, version 8.4) was utilized to render and obtain 3D reconstructed images using the “surface tool”. After reconstruction, a digital zoom tool was used for image magnification, as shown in Fig. 7H (right) and I. GluA1^+^ puncta were rendered using the “Spot Tool”. Distributed puncta inside microglia were defined as engulfed synaptic puncta. The distance is less than 0 μm from the microglial surface and contacted puncta distributed outside microglia, but the distance is less than 0.25 μm from the microglial surface.

### Flow cytometry

For peripheral blood mononuclear cells (PBMC) analysis, 200 µL whole blood sample from another set of APP/PS1, APP/PS1 + Serum, and APP/PS1 + Ly6G mice was collected into 2-mL centrifuge tubes containing 1% heparin sodium (10 μL). After anticoagulation, the solution was diluted with equal sterile saline. PBMCs were isolated using ammonium-chloride-potassium (ACK) lysing buffer according to the manufacturer’s instructions. The animals were perfused with cold sterile saline to remove the remaining peripheral blood, and the brains were harvested. The method for the generation of brain immune cells was used in a previous study with slight modification [31]. The brain was rapidly dissected and immersed in cold Hank’s balanced salt solution (HBSS). The brain was minced by scissors and homogenized using a Dounce homogenizer in ice-cold HBSS 30 times without bubbles. The cell suspension was then transferred to a prechilled 15-mL tube and filtered through a prewet (HBSS) 70-μm nylon mesh. Myelin and debris were removed using a modified cold 40% Percoll (Merck Millipore, 17-0891-02) gradient by centrifugation for 30 min at 500 × *g* with no acceleration or braking. The pellet of immune cells contained microglia, monocytes, and neutrophils at the bottom of a 15-mL tube. The samples were labeled with FITC-conjugated anti-CD45 (0.25 mg/10^6^ cells; 11-0451-81, eBioscience), PE-Cyn7-conjugated anti-CD11b (0.125 mg/10^6^ cells; 25-0112-81, eBioscience), APC-conjugated anti-CD62L (0.5 mg/10^6^ cells; 553152, B&D), FITC-conjugated anti-CXCR4 (0.5 mg/10^6^ cells; 551967, B&D), PE-conjugated anti-Ly6G (0.5 mg/10^6^ cells; E-AB-F1108D, Elabscience), EF450-conjugated anti-Ly6C (0.5 mg/10^6^ cells; 48-5932-80, Invitrogen), PE-conjugated anti-CD31 (0.25 mg/10^6^ cells; B&D, 553373, MEC 13.3), BB700-conjugated anti-VEGFR2/FLK-1 (0.25 mg/10^6^ cells; 742184, B&D) and FITC-conjugated anti-CXCL1 (0.6 mg/10^6^ cells; IC4532G, B&D) antibodies at a 1:100 dilution for 30 min on ice. The experimental samples were analyzed using a flow cytometer (Beckman CytoFLEX S, CA, USA).

### Cytokine antibody array

The cytokine profiles of the serum of the WT + PBS, WT + Serum, APP/PS1 + PBS, and APP/PS1 + Serum groups (*n* = 1 mixture of four serum samples) were analyzed using a mouse cytokine antibody array (CAT # QAM-CAA-4000; RayBiotech, ATL, USA) and tested according to the experimental protocols. A combination of factors, including ranking by fold change (<0.67 and >1.5) and signal intensity (>150), was used to identify robust changes, and a meta-ranking of proteins was generated. The relative signal intensity of the indicated cytokines and Gene Ontology (GO) functional enrichment data are presented in a trend graph. The detailed protocol of the assay using a Quantibody® mouse cytokine antibody array 4000 has been described previously [32].

### Neutrophil depletion

The neutrophil depletion procedures have previously been described [4, 10]. Neutrophils were depleted 24 h prior to the first serum injection by i.p. injection of 0.3 mg of anti-mouse Ly6G antibody or isotype control (In*Vivo*Plus anti-mouse Ly6G, 1A8; BE0075-1, Bioxcell, NH, USA) every 3 days. Anti-ly6G antibody and serum injections were continued for 4 weeks until behavioral tests. Peripheral neutrophil levels remained very low, which was confirmed by immunofluorescence assay after behavioral tests. The neutrophil procedure was continuous to eliminate potential rebound effects.

### Enzyme-linked immunosorbent assay (ELISA)

The concentrations of CXCL1, CCL3, CXCL16, CXCL9, HGF, FetuinA, EGF, FGF2, IL-22, TGFB1, and VEGF-A (Beijing 4A Biotech) in both serum and the hippocampus were measured as previously described [29, 30]. Blood samples were centrifuged at 4 °C (at 4000 × *g* for 15 min), and sera were transferred to another set of tubes. Hippocampi were homogenized in RIPA lysis buffer (20 mM Tris-HCl (pH 8.0), 137 mM NaCl, 1% NP40, 10% glycerol, 1 mM PMSF, 1 μL/mL aprotinin, 1 μg/mL leupeptin, and 0.5 mM sodium vanadate).

### Cell line and cell culture

Mouse brain microvascular endothelial cells (MBECs, bEnd.3, Procell, CL-0598, Wuhan, China) were cultured according to the manufacturer’s instructions. Briefly, MBECs were cultured in 10% fetal bovine serum/Dulbecco’s modified Eagle’s medium (DMEM) and 1% penicillin-streptomycin (Life Technologies, Inc.) in uncoated T75 flasks at 37 °C and 5% CO_2_. Full media changes were performed every other day. MBECs were passaged using 0.25% trypsin treatment for 1 min at 37 °C. A total of 10,000 cells/cm^2^ were cultured in serum-free medium for 24 h and then treated with serum (20 μL), anti-VEGF-A antibody (2 μg) and VEGF-A protein (20 ng/mL) for 24 h (CME0014, Beijing 4A Biotech).

### CRISPR-Cas9 knockout

For CRISPR/Cas9 knockout of the murine *Cdk5* gene in bEnd.3 cells, the small guide RNAs (sgRNAs) were used: sgCDK5#1: CAGGCTGGATGATGACGATG; sgCDK5#2: CCGGGAAACTCATGAGATTG. sgRNA sequences were verified in NCBI online tool (https://www.ncbi.nlm.nih.gov). The sgRNA sequences were cloned into our previously constructed vector pSB-CRISPR as previously described [33]. The efficiency of pSB-CRISPR vector tools was identified by Western blotting with a specific anti-Cdk5 antibody.

### Western blot analysis

Total proteins were extracted from cultured bEnd.3 cells and brain tissues using protein lysis buffer (P0013C, Beyotime), containing 1% phosphatase inhibitors (Sigma-Aldrich), and 1% protease inhibitor cocktail (Sigma-Aldrich) and centrifuged at 12,000 × *g* (30 min, 4 °C). The total protein concentration was determined using a BCA protein assay kit (Beyotime, P0012) and was adjusted to 3.5 mg/mL. A total of 30 μg of protein was separated by 4–10% SDS-PAGE and transferred to a polyvinylidene fluoride (PVDF) membrane. The membrane was blocked with Tris-buffered saline with Tween-20 (TBST) containing 5% milk and analyzed using monoclonal antibodies against Ki67 (1:2,000, SolA15; eBioscience), Cdk5 (1:2,000, DC 17; Santa Cruz), p-Cdk5 (1:2,000, C-7; Santa Cruz), mouse anti-glutamate receptor 1 (1:2000; ab183797, Abcam), mouse anti-synaptophysin (1:2000; S5768, Sigma-Aldrich), mouse anti-PSD95 (1:2000; ab13552, Abcam), total Tau (D1M9X, 1:2000, 46687, Cell Signaling Technology), phospho-Tau Ser^199/202^ (1:2000, AB9674; Sigma-Aldrich) and rabbit anti-β-actin (1:2,000, 4970; Cell Signaling Technology). Blots were scanned using Image Quant Las4000mini System. ImageJ software (NIH) was used for the mean intensity of bands.

### VEGF-A and AAV injections

APP/PS1 mice were injected intraperitoneally with 2 μg/kg/d recombinant mouse VEGF-A or saline for 3 days as described previously with slight modification [34]. Systematic recombinant AAV-BR1-CMV-mCdk5-P2A-EGFP (BR1-mCdk5) or AAV-BR1-CMV-EGFP (BR1-CON) was injected into 30-week-old APP/PS1 mice (7.5 months old) through the tail vein (mCdk5; 1.0 × 10^12^ genomic particles/mouse). Further treatment, such as serum injection, was performed 2 weeks after AAV injection. AAV was produced by a triple plasmid transfection system according to the previous studies [35]. In brief, one plasmid contains capsid BR1, a second plasmid comprises the ITR-flanked Cdk5 gene, and a third plasmid provides helper genes.

### Isolation of brain endothelial cells from the hippocampus

Primary endothelial cells in the hippocampus were isolated as previously described [36]. Briefly, hippocampi of APP/PS1 mice treated with BR1-mCdk5 or BR1-CON were minced and digested for 30 min at 37 °C with 2% (vol/vol) FBS in PBS containing 400 U/mL collagenase. After enzymatic digestion, the tissue was pelleted by centrifugation at 300 × *g* at 4 °C for 5 min, and the supernatant was discarded. Cell pellets were resuspended in 2% (vol/vol) FBS in PBS containing DNase I (0.5 mg/mL), and single-cell suspensions were gently added on top of 22% Percoll and centrifuged at 560 × *g* at 4 °C for 10 min to obtain the vascular cells at the bottom of the tube. Purified cells were analyzed on a FACS Calibur flow cytometer (BD influxTM, NJ, USA) using an anti-CD31-PE antibody (0.25 mg/10^6^ cells; B&D).

### RNA isolation and real-time qPCR

Mouse hippocampal endothelial cells were isolated as described above [36]. Total RNA from the hippocampal endothelial cells was isolated using RNAiso Plus (Sangon Biotech, Shanghai, China). cDNA was synthesized using a GoScriptTM cDNA Reverse Transcription Kit (Promega, Madison, WI, USA). The expression of specific mRNAs was assayed using fluorescence-based real-time quantitative PCR (RT-PCR). Quantitative PCRs were performed using TransStart Tip Green qPCR SuperMix (TransGen Biotech, Beijing, China) in triplicate for each sample. The details of the primer sequences are as follows:

Cdk5 (forward), 59-CAATGCAGAAATACGAGAAACTGG-39;

Cdk5 (reverse), 59-CTTTGAGTAGACAGATCTCCCG-39;

Cxcl1 (forward), 59-ACCGAAGTCATAGCCACACTC-39;

Cxcl1 (reverse), 59-CTCCGTTACTTGGGGACACC-39.

### Evans blue leakage analysis

Blood-brain barrier (BBB) permeability was assessed using Evans blue staining, as previously described with slight modifications [30]. Briefly, a 2% solution of Evans blue dye (E2129; Sigma-Aldrich) in 0.9% saline was intravenously injected into the APP/PS1 mice via the tail vein at a dose of 2 mL/kg one day after the last VEGF-A injection. Two hours later all of the mice were anesthetized and transcardially perfused with cold 0.9% saline. To detect Evans blue leakage, brain tissue samples were homogenized in 99% dimethylformamide and incubated in a 50 °C water bath for 48 h. The supernatant was collected after centrifugation at 12,000 × *g* (30 min, 4 °C), and the absorbance of the sample was measured at 620 nm using a microplate reader.

### Electrophysiology

Coronal brain slices from the hippocampus (300 μm thick) were prepared from postnatal 5–6-month-old male APP/PS1 mice and their WT littermates using a vibratome (Leica VT1000) in a pre-chilled solution containing (in mM) 110 choline chloride, 7 MgCl_2_·6H_2_O, 2.5 KCl, 0.5 CaCl_2_·H_2_O, 1.3 NaH_2_PO4, 25 NaHCO_3_, 20 glucose, saturated with 95% O_2_ and 5% CO_2_. The slices were immediately transferred to artificial cerebrospinal fluid (ACSF) at 35 °C for 30 min then transferred to a recording chamber. The recording of ACSF containing (in mM): 125 NaCl, 2.5 KCl, 2 CaCl_2_·H_2_O, 1.3 MgCl_2_·6H_2_O, 1.3 NaH_2_PO4, 25 NaHCO_3_, and 10 glucose. All ACSF solutions must be saturated with carbogen (95% O_2_/5% CO_2_) prior to use to ensure stable pH buffering (pH 7.3) and adequate oxygenation. Whole-cell patch-clamp recordings from the soma of dentate gyrus (DG) pyramidal neurons were obtained under an infrared (IR)-differential interference contrast (DIC) microscope (ECLIPSE FN1, Nikon). To record mEPSC, the holding potentials were held at −70 mV, the GABA_A_ receptor and action potentials were blocked with 20 µM bicuculline methiodide (BMI) and 1 µM tetrodotoxin (TTX), respectively. Glass pipettes were filled with an internal solution containing (in mM) 100 CsMeSO_4_, 25.5 CsCl, 10 HEPES, 8 NaCl, 0.25 EGTA, 10 glucose, 4 MgATP and 0.3 Na_3_GTP (pH 7.3, 285 mOsm). Data were digitized at 10 kHz and filtered at 1 kHz and recorded using a multiClamp 700B amplifier and Digidata 1500B with pClamp11.2 software (Molecular Devices). Data were collected when the series resistance fluctuated within 20% of the initial values and analyzed using the Mini Analysis Program (Synaptosoft) and pClamp 11.2 software (Molecular Devices). The method of electrophysiology was performed according to the studies previously reported [37, 38].

### Hematological analysis

A total of 100 µL of whole blood samples from wide-type, APP/PS1 mice and APP/PS1 mice injected with serum were collected by the heart puncture into heparin-coated tubes. All peripheral blood samples were analyzed with an automatic hematology analyzer (Sysmex, XT-2000i, Sysmex Corporation, Japan). The following blood parameters were analyzed: white blood cell count (WBC), red blood cell count (RBC), neutrophil (Neu), lymphocyte (Lym), monocyte (Mon), eosinophil (Eos), basophil (Bas), hemoglobin (HGB), hematocrit (HCT), mean corpuscular volume (MCV), mean corpuscular hemoglobin (MCH), mean corpuscular hemoglobin concentration (MCHC), platelet count (PLT), mean platelet volume (MPV), platelet distribution width (PDW).

### Statistical analysis

All results represent at least three independent experiments and are expressed as mean ± SEM. An unpaired two-sided Student’s *t* test (between two groups) or one-way or-two-way ANOVA (Bonferroni or LSD comparison tests) or Kolmogorov–Smirnov test was performed for statistical analysis. Two-way repeated ANOVA was performed in MWM test results. One-way ANOVA (equal homogeneity of variance) or Dunnett T3 (unequal homogeneity of variance) was used for hematologic profile analysis. Data distribution was assumed to be normal; this was also tested with QQ-plots or histograms. Analysis and graphing were performed using GraphPad Prism version 8.0 (GraphPad 8.0). *P* < 0.05 was considered statistically significant.

## Results

### Wild-type serum injection alleviates hyperreactive neutrophil infiltration in the brains of APP/PS1 mice

**Fig. 1:**
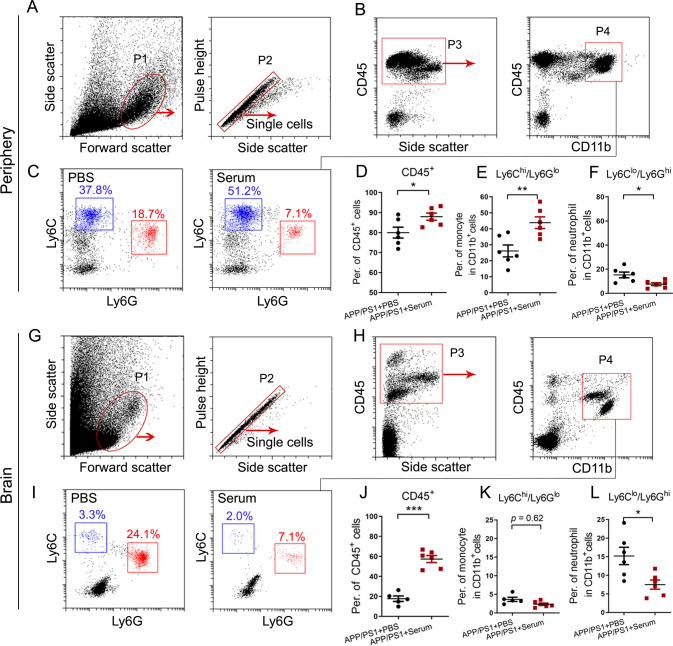
Wild-type serum influences peripheral and brain innate immune subsets in APP/PS1 mice. Flow cytometry sorting gating strategy of immune cells (P1), single cells (P2), CD45^+^ leukocytes (P3), and CD45^hi^/CD11b^+^ monocytes (P4) in PBMCs (**A**, **B**) and the brain (**G**, **H**). The red arrows in **A**-**B** and **G**-**H** represent the P2 subset based on P1, the P3 subset based on P2, and the P4 subset based on P3. **C** Monocytes have distinctly different populations based on Ly6C and Ly6G expression. **D**–**F** Flow cytometry analysis of CD45^+^ leukocytes (P3), Ly6C^hi^/Ly6G^lo^ monocyte (indicated by the blue gate in **C**), and Ly6G^hi^/Ly6C^lo^ neutrophil (indicated by the red gate in **C**) frequencies in the PBMCs of PBS- and serum-treated APP/PS1 mice. **I** Monocytes have distinctly different populations based on Ly6C expression. **J**–**L** Flow cytometry analysis of the frequencies of CD45^+^ leukocytes (P3), Ly6C^hi^/Ly6G^lo^ monocytes (indicated by the blue gate in **I**), and Ly6C^lo^/Ly6G^hi^ neutrophils (indicated by the red gate in **I**) in the brains of PBS- and serum-treated 8–9-month-old APP/PS1 mice (data are presented as the mean ± s.e.m.; unpaired Student’s two-tailed *t* test, *p < 0.05; **p < 0.01; ***p < 0.001; *n* = 5–6 per group).

**Fig. 2:**
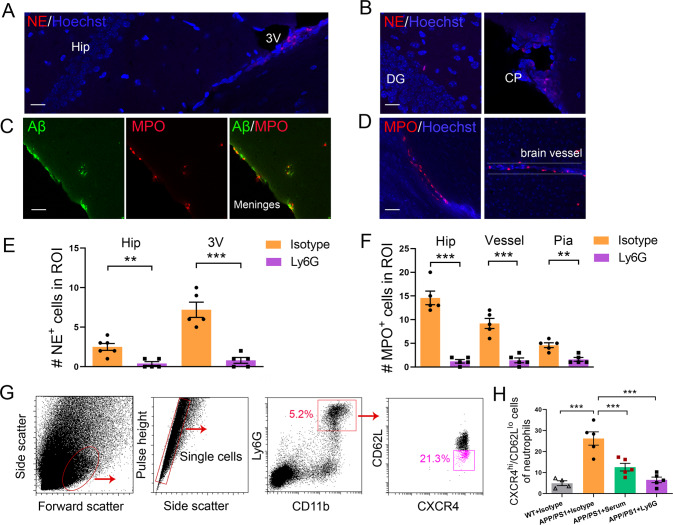
Neutrophils migrate into the brain during disease progression in APP/PS1 mice. Representative confocal images of NE^+^ leukocytes (red cells) in the 3V (**A**), around the hippocampal area (left panels in **B**), and the choroid plexus (right panels in **B**). MPO^+^ leukocytes (red cells) in the meninges and parenchyma where Aβ plaque (green staining) is deposited (**C**) and around brain vessels (right panels in **D**) in 8- to 9-month-old APP/PS1 mice. The brain vessels were identified based on Hoechst staining 3D using Zen software. Quantification of the number of NE^+^ (**E**) and MPO^+^ (**F**) cells in the hippocampus, 3V, blood vessels and pia mater in APP/PS1 mice treated with anti-Ly6G antibody or isotype control antibody (data are presented as the mean ± s.e.m.; unpaired Student’s two-tailed *t* test, *n* = 5–6; **p < 0.01; ***p < 0.001). **G**, **H** Flow cytometry analysis of hyperreactive neutrophils infiltrating the brain, as reflected by CXCR4^hi^/CD62L^lo^ staining in APP/PS1 mice treated with serum or anti-Ly6G antibody and WT controls (*n* = 4–5). One-way ANOVA followed by Bonferroni multiple comparison tests. ***p < 0.001. Hip hippocampus, 3V third ventricle, DG dentate gyrus, CP choroid plexus, Scale bar: 20 μm in **A** and **B**; 50 μm in **C** and **D**.

Innate immunity is associated with AD pathology [39–41]. To investigate innate immune cell response to wild-type serum treatment in APP/PS1 mice, we used flow cytometry to analyze the activities of CD45^+^ and CD45^+^/CD11b^+^ immune cell subsets in peripheral blood mononuclear cells (PBMCs, Fig. 1A, B; Supplementary Fig. 1A, B) and brain cell suspensions (Fig. 1G, H). Interestingly, following the serum treatment, there was an increase in the numbers of CD45^+^ immune cells, but a decrease in CD11b^+^Ly6G^hi^Ly6C^lo^ neutrophils, both in the peripheral blood (Fig. 1C, D, F) and the brain (Fig. 1I, J, L) in the disease model. Notably, serum injection significantly elevated the cell count of CD11b^+^/Ly6C^hi^/Ly6G^lo^ monocytes (Fig. 1C, E), which is consistent with the results reflected by CD11b^+^/CD45^hi^ (Supplementary Fig. 1B, C). Unexpectedly, we observed inconsistent results between the numbers of CD11b^+^/Ly6C^hi^/Ly6G^lo^ monocytes (Fig. 1I, K) and CD11b^+^/CD45^hi^ monocytes (Supplementary Fig. 1E, F) in serum-treated APP/PS1 mouse brains. Given that neutrophils can produce neutrophil extracellular traps (NETs), we analyzed the presence of NETs in the brains of AD mice by anti-neutrophil elastase (NE) or anti-myeloperoxidase (MPO) immune fluorescent staining. Indeed, we observed neutrophils accompanied by NETs in the parenchyma, which are primarily localized around the cerebral ventricle, choroid plexus, leptomeninges, Aβ plaque and cortical vessels (Fig. 2A–D). Consistently, Ly6G antibody treatment abolished NE^+^ or MPO^+^ neutrophils adjacent to the hippocampus, ventricular spaces and leptomeninges in AD mice (Fig. 2E, F). Next, we investigated the phenotype of infiltrating neutrophils in an AD mouse model by flow cytometry analysis. The number of harmful hyperreactive Ly6G^+^/CXCR4^hi^/CD62L^lo^ senescent neutrophil subsets was significantly increased in APP/PS1 mouse brains compared with WT controls, whereas these effects were prevented by serum or Ly6G neutralizing antibody treatment (Fig. 2G, H). Together, these results suggest that serum injection in AD mice decreased the number of peripheral neutrophils and prevented hyperreactive neutrophil subset migration to the brain.

### Wild-type serum injection alters peripheral immune profiles associated with leukocyte migration and chemotaxis in APP/PS1 mice

**Fig. 3:**
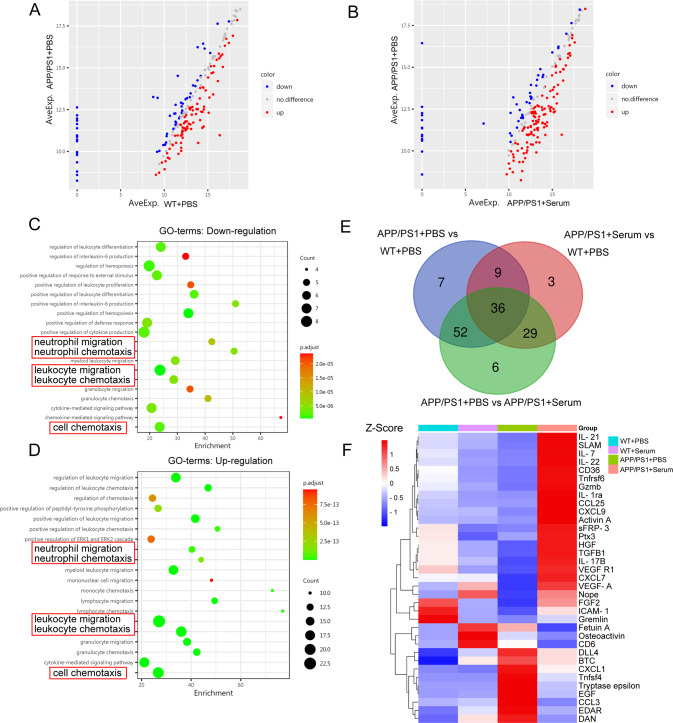
Wild-type serum injection influences peripheral immune profiles in APP/PS1 mice. Mouse cytokine antibody array analysis was performed in the serum of PBS- or serum-treated APP/PS1 mice (8–9 months old) and PBS- or serum-treated age-matched WT mice. **A**, **B** Downregulated (blue) and unregulated (red) cytokine expression was detected in the serum between WT + PBS and APP/PS1 + PBS mice and between APP/PS1 + PBS and APP/PS1+Serum mice. Gene Ontology enrichment analysis (biological process) of 23 downregulated cytokines (**C**) and 59 upregulated cytokines (**D**) in APP/PS1+Serum *vs*. APP/PS1 + PBS groups. **E** Venn diagram showing the overlapping cytokines in the serum of the four groups. Blue represents the APP/PS1 + PBS *vs*. WT + PBS groups; red represents the APP/PS1 + Serum *vs*. WT + PBS groups; and green represents the APP/PS1 + Serum *vs*. APP/PS1 + PBS groups. **F** Unsupervised clustering gene expression heatmap analysis showing the 36 overlapping cytokines among the serum of WT + PBS, WT + Serum, APP/PS1 + PBS, and APP/PS1 + Serum mice.

The circulating immune microenvironment plays an important role in cerebral homeostasis in AD [40–42]. We showed that serum injection reduced the number of peripheral and cerebral neutrophils in APP/PS1 transgenic mice. However, how reduced peripheral neutrophils may attenuate their infiltration into the parenchyma in AD mice remains unclear. Therefore, we first analyzed the cytokine profiles of the serum of WT + PBS, WT + Serum, APP/PS1 + PBS, and APP/PS1 + Serum mice using a mouse cytokine array. Unsupervised hierarchical clustering based on differentially expressed proteins (DEPs) indicated a distinct protein expression network induced by serum (Fig. 3A, B). The top 82 ranked proteins are listed in Supplementary Table 1. Gene Ontology (GO) analysis showed significant enrichment of proteins mainly involved in neutrophil migration, leukocyte migration, and chemotaxis (Fig. 3C, D). Kyoto encyclopedia of genes and genomes (KEGG) analysis using the top 82 ranked proteins (23 downregulated and 59 upregulated) revealed a network of cytokine-cytokine receptor interactions and chemokine signaling pathways (Supplementary Fig. 2A, B).

**Fig. 4:**
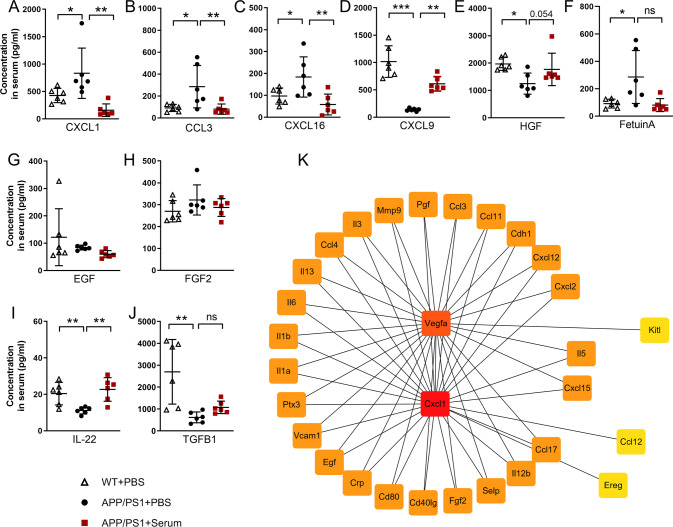
The levels of cytokines measured in the serum of WT + PBS, APP/PS1 + PBS, and APP/PS1 + Serum mice. Our data showed that the levels of CXCL1 (**A**), CCL3 (**B**), CXCL16 (**C**), and FetuinA (**F**) were significantly increased, and the levels of CXCL9, HGF, IL-22 and TGFB1 were significantly decreased in the serum of APP/PS1 mice (8–9 months old) relative to those in the age-matched WT mice (**D**, **E**, **I** and **J**). However, these increased alterations were restored to the levels detected in WT + PBS mice after serum treatment. No obvious differences in the levels of EGF and FGF2 were noted in the serum of WT + PBS, APP/PS1 + PBS, and APP/PS1+Serum mice (**G**, **H**). **K** Protein-protein interaction (PPI) network based on the online tool STRING using the top 82 ranked proteins produced the strongest network around chemotactic cytokine and angiogenic factor signaling (CXCL1 and VEGF-A). Mean concentrations are expressed in pg/mL of serum. One-way ANOVA followed by Bonferroni multiple comparison tests. Data are presented as the mean ± s.e.m.; ns nonsignificant; *n* = 6 per group; *p < 0.05; **p < 0.01; ***p < 0.001.

Furthermore, 36 DEPs (Supplementary Table 2) in the serum of WT + PBS, WT + Serum, APP/PS1 + PBS, and APP/PS1 + Serum mice were identified by a Venn diagram (Fig. 3E) and heatmap (Fig. 3F). Chemokines, growth factors and cytokines were identified from the 36 DEPs, including CXCL1, CCL3, CXCL16, CXCL9, HGF, FetuinA, EGF, FGF2, IL-22, and TGFB1. These factors are significantly enriched in neutrophil/leukocyte migration and chemotaxis. We further confirmed the results using ELISA kits in the serum and brain of WT + PBS, APP/PS1 + PBS, and APP/PS1 + Serum mice (Fig. 4A–J).

Consistent with the results of the cytokine antibody array test, the levels of the CXCL1, CCL3, and CXCL16 chemokines associated with neutrophil migration were significantly increased in the brains of APP/PS1 + PBS mice. After 4–6-week-old wild-type serum treatment, the increased expression of these three chemokines was normalized in both the periphery and the brain, reaching the levels of age-matched (9 months old) WT mice (Fig. 4A–C; Supplementary Fig. 3A–C). Notably, ingenuity pathway analysis (IPA) using the top-ranked proteins produced the strongest network around CXCL1 and VEGF-A signaling (Fig. 4K). Moreover, we performed the test using a hematology analyzer in wide-type mice, APP/PS1 mice treated with or without serum. Consistent with the data in flow cytometry tests (Fig. 1E; Fig. S1C), hematological data showed that serum injection significantly decreased the number of neutrophils and increased the monocyte counts (Supplementary Table 3). These results indicate that serum-derived systemic circulating chemokines may modulate proinflammatory neutrophil infiltration into the brain in an AD mouse model.

### Wild-type serum injection blocks CXCL1 secretion via endothelial VEGF/Cdk5 signaling

**Fig. 5:**
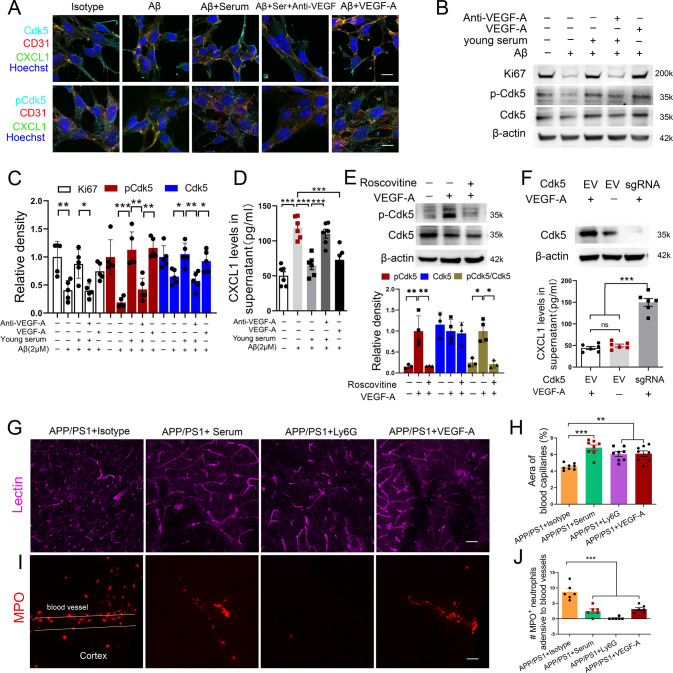
Wild-type serum-derived VEGF-A attenuates capillary loss and mediated endothelial Cdk5 signaling inhibiting CXCL1 secretion. **A** Representative images of CDK, pCDK, and CXCL1 expression in bEnd.3 cells (CD31^+^). Scale bar: 10 μm. **B** Western blotting (WB) for cell proliferation markers Ki67, phosphorylated Cdk5, and Cdk5, in bEnd.3 cells were treated with Aβ (2 μM), serum (20 μL), anti-VEGF-A antibodies (2 μg), and recombinant VEGF-A protein (20 ng/mL). **C** Quantification of the band for Ki67, pCdk5, and Cdk5 proteins using Western blot analysis; *n* = 5 per group. **D** ELISA analysis for CXCL1 levels related to **C** in the supernatant; *n* = 6 per group. **E** Cdk5 inhibitor roscovitine (20 μM) inhibits VEGF-A-induced Cdk5 phosphorylation in bEnd.3 cells; *n* = 3–4 per group. **F** Effect of CDK5 knockout using a sgRNA on CXCL1 expression in bEnd.3 cells with or without VEGF-A stimulation; *n* = 6 per group. **G**, **H** Representative images and quantification of cerebral angiogenesis (lectin^+^) in WT, APP/PS1 mice, APP/PS1 mice treated with anti-Ly6G antibodies or VEGF-A recombinant protein; *n* = 8 mice per group, 12 image fields from 5 brain slices per animal were analyzed and averaged as a value; Scale bar: 50 μm in **G**. **I**–**J** Representative images and quantification of neutrophil adhesion stained by anti-MPO antibody in cortical capillaries in APP/PS1 mice (white bilinear; the vessels identified based on the Z-Stack tool); one-way ANOVA followed by Bonferroni multiple comparison tests. ns nonsignificant; *n* = 6 mice per group, 10 image fields from 4 brain slices per animal were analyzed and averaged as a value; *p < 0.05; **p < 0.01; ***p < 0.001 for all. Scale bar: 20 μm in **I**. The three separate in vitro experiments that produced comparable outcomes are shown by the data.

We next investigated the molecular mechanism underlying neutrophil adhesion/infiltration in APP/PS1 mice. We focused on the cytokine CXCL1, a neutrophil attractant chemokine in ischemic stroke [43], multiple sclerosis [44] and multiple murine models of neuroinflammation [45–47]. Endothelial-specific Cdk5 knockout increased CXCL1 expression and led to progressive astrogliosis in epilepsy [48]. Consistent with the previous study, we found a negative relationship between CXCL1 levels and Cdk5/pCdk5 levels in bEnd.3 cells after Aβ or serum treatment (Fig. 5A; Supplementary Fig. 4A, B). Colocalization analysis confirmed that Cdk5, pCdk5 and CXCL1 proteins existed in the cytoplasm of brain microvascular endothelial cells (Supplementary Fig. 4C, D). However, the Cdk5 and pCdk5 distribution obviously differed from the CXCL1 distribution in the cytoplasm (Supplementary Fig. 4E, F).

Additionally, we found that VEGF-A was another key molecule by IPA after serum treatment (Fig. 4K), and serum exposure increased circulating VEGF-A levels in APP/PS1 mice (Supplementary Fig. 5). Additionally, VEGF-A signaling modulates brain functions in aging progression [22]. Inhibition or silencing of Cdk5 reduced endothelial cell migration and angiogenesis by interfering with the actin cytoskeleton [49, 50]. Consistently, our data showed that decreased endothelial cell proliferation paralleled the low expression of Cdk5 after Aβ treatment in vitro (Fig. 5B, C), implying a potential link between Cdk5 and angiogenesis. Therefore, we hypothesize that VEGF-A may affect CXCL1 expression via Cdk5 signaling. First, we found that serum failed to prevent the Aβ-induced decreases in Cdk5 expression and phosphorylation in bEnd.3 cells after VEGF-A antibody treatment (Fig. 5B, C). Similar to the beneficial effects of the serum, VEGF-A also significantly prevented the Aβ-induced increase in CXCL1 expression (Fig. 5D). To further study how VEGF-A mediated low CXCL1 expression, we examined Cdk5 activity by using roscovitine, an inhibitor of Cdk5 (Fig. 5E). We found that roscovitine attenuated the inhibitory effect of VEGF-A on CXCL1 expression (Supplementary Fig. 6A–G). In line with these results, knockout of the endothelial *Cdk5* gene in bEnd.3 cells via CRISPR-Cas9 gene editing also prevented VEGF-A-mediated CXCL1 suppression, instead inducing a threefold increase in CXCL1 levels in the supernatant (Fig. 5F).

Neutrophil adhesion in brain capillaries reduces cortical blood flow, which results in vascular abnormalities and exacerbates brain pathology in multiple mouse models of Alzheimer’s disease [10, 11]. We observed that serum promoted angiogenesis and decreased capillary segments in APP/PS1 mice (Fig. 5G, H). To investigate the role of neutrophil adhesion in cortical capillaries, we administered Ly6G neutralizing antibodies to deplete peripheral neutrophils in APP/PS1 mice. Our results showed that the Ly6G antibody mimicked the beneficial effects of serum by increasing the number of capillaries (Fig. 5G, H). Indeed, Ly6G antibody treatment led to an approximate 90% reduction in the number of MPO^+^ neutrophils adjacent to cortical capillaries (Fig. 5I, J; Fig. 2D, F). These data suggest that serum may increase brain capillary density by preventing neutrophil adhesion/infiltration.

We next tested the hypothesis that serum-derived VEGF-A was necessary for the blockade of neutrophil adhesion and brain capillary loss in vivo. Therefore, we applied recombinant VEGF-A protein to APP/PS1 mice and found that VEGF-A could promote Cdk5 and pCdk5 activity (Supplementary Fig. 7A–F, I–N), increase brain angiogenesis and decrease tiny capillary segments (Fig. 5G, H), followed by a decrease in neutrophil adhesion in APP/PS1 mice (Fig. 5I, J). Similarly, the Ly6G neutralizing antibody exhibited similar benefits to serum and VEGF-A protein on the number of capillaries and neutrophil adhesion adjacent to cortical capillaries (Fig. 5I, J). Notably, we observed no additional worsening of the BBB permeability and integrity in the brains of VEGF-A-treated APP/PS1 mice as compared to PBS-treated APP/PS1 mice (Supplementary Fig. 8A–C). Together, these results indicate that serum-derived VEGF-A suppresses CXCL1 expression via Cdk5 activity, which results in the inhibition of neutrophil recruitment into the AD mouse brain.

### Administration of Ly6G antibody improves learning and memory

Next, to determine the role of migratory neutrophils in the brain in cognitive impairment, we assessed spatial learning and memory performance using contextual fear conditioning and Morris water maze tests. During fear conditioning training, all groups showed comparable baseline freezing time (Supplementary Fig. 9A). However, we found that freezing behaviors were significantly increased during the contextual (Supplementary Fig. 9B), but not cued (Supplementary Fig. 9C) fear memory test in APP/PS1 mice following neutrophil depletion. The performance was similar to that of WT mice and serum-treated APP/PS1 mice (Supplementary Fig. 9B). In the MWM test, administration of Ly6G antibody in APP/PS1 mice also improved performance for hidden platform location during the acquisition phase (Supplementary Fig. 9D). During the spatial probe phase, APP/PS1 mice with neutrophil depletion spent more time and traveled greater distances in the target quadrant (Supplementary Fig. 9E, F; increases of 46.7% and 53.4% for time and distance in the quadrant, respectively) and had a significantly greater number of passes across the platform than the APP/PS1 mice (Supplementary Fig. 9G; 2-fold increase). Additionally, we observed no obvious differences in the swimming speed of all groups (Supplementary Fig. 9H). The deletion efficiency in the peripheral blood, brain and meninges was confirmed by flow cytometry analysis and immunofluorescence staining (Fig. 2A–F; Supplementary Fig. 10A–F; Supplementary Fig. 11A–E). However, administration of Ly6G antibody had no influence on the percentage of CD3^+^ T lymphocytes (Supplementary Fig. 11F–I), or the health conditions in APP/PS1 mice, as reflected by exploratory abilities and velocity of movement in the OFT (Supplementary Fig. 11J–L). These results demonstrate that treatment with Ly6G antibody has beneficial effects on memory behavior in APP/PS1 mice.

### Endothelial Cdk5 overexpression mitigates neutrophil infiltration and improves cognitive function

**Fig. 6:**
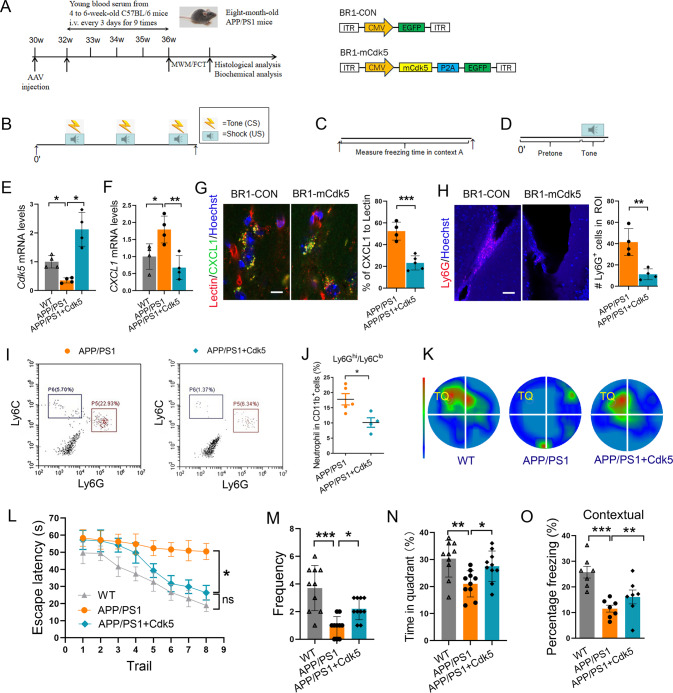
Endothelial Cdk5 overexpression mitigates neutrophil infiltration and improved cognitive function in APP/PS1 mice. **A** Scheme of the experimental details. Systematic AAV-mCdk5 expression was determined in 30-week-old (around 7.5 months old) APP/PS1 mice. After 2 weeks (8 months old), AAV-treated male APP/PS1 transgenic mice were intravenously injected 9 times with 200 μL serum from 4-week-old C57BL/6 mice every 3 days. Behavioral analyses, including fear conditioning tests (FCT) and the MWM test, were performed after the last serum injection. Schematic representation of AAV2-BR1 constructs indicating the inverted terminal repeats (ITRs) at both ends and CMV promoter-driven mCdk5 with EGFP (BR1-mCdk5) or CMV promoter-driven EGFP (BR1-CON). **B**–**D** Schematic diagram of the fear conditioning procedure. **B** Training day (context I); **C** Contextual test day (context I); **D** Cued test day (context II). **E**, **F** The relative mRNA levels of *Cdk5* and *CXCL1* in primary endothelial cells from the hippocampi were evaluated in BR1-mCdk5 and BR1-CON-injected APP/PS1 mice at 32 weeks of age (*n* = 4 per group). Representative confocal images of CXCL1 expression in cerebral microvessels (green, **G**) and neutrophils (red, **H**) in the lateral ventricles of APP/PS1 mice injected with BR1-mCdk5 or BR1-CON via the tail vein. Scale bar: 10 μm in **G**; 50 μm in **H**. **I**, **J** Representative flow cytometry analysis and quantification of neutrophils (Ly6G^hi^/Ly6C^lo^) in the brains of APP/PS1 mice injected with BR1-Cdk5 or BR1-CON (*n* = 4–5 mice per group). **K** Representative heatmap of escape trajectory in probe phase of the MWM task. **L** BR1-mCdk5 or BR1-CON-injected APP/PS1 mice (8 months old) and age-matched WT mice were assessed in the MWM task. **M**, **N** The frequency and time spent in the target quadrant (TQ) in the groups. **O** Contextual freezing test. *n* = 7 or 10 mice per group for the behavioral test. All data are shown as the mean ± s.e.m.; two-way repeated ANOVA, Bonferroni multiple comparison tests; *p < 0.05; **p < 0.01; ***p < 0.001.

To further determine the role of Cdk5 signaling in neutrophil infiltration and memory deficits in the APP/PS1 brain, we used AAV2-BR1-CMV-mCdk5-P2A-EGFP (BR1-mCdk5) to induce brain endothelial Cdk5 overexpression and carried out the behavioral test according to the experimental scheme (Fig. 6A–D). RT-PCR confirmed that *Cdk5* mRNA was increased in brain endothelial cells after AAV delivery (Fig. 6E, F). Similar increases in Cdk5 protein levels were observed in the hippocampus using immunofluorescent staining (Supplementary Fig. 12A, B).

Consistent with the in vitro results, CXCL1 expression in brain vessels was decreased after Cdk5 overexpression in APP/PS1 mice, as shown by immunofluorescent staining (Fig. 6G). In addition, overexpression of endothelial Cdk5 resulted in a decrease in migratory neutrophils to the brain (Fig. 6H–J), shortened the escape latency (Fig. 6L), increased the frequency of passes across the platform (Fig. 6K, M) and the duration in the target quadrant in the MWM test (Fig. 6N), and increased freezing time in contextual but not cued memory test (Fig. 6O). Thus, these results are largely consistent with the in vitro data and demonstrate that Cdk5 overexpression decreases CXCL1 secretion in brain endothelial cells.

Together, our results support that enhanced Cdk5 signaling inhibits neutrophil infiltration and rescues memory deficits, which suggests the beneficial effects of serum treatment in an AD mouse model.

### Inhibition of neutrophil infiltration by wild-type serum rescues synaptic loss and microglial activation in APP/PS1 mice

**Fig. 7:**
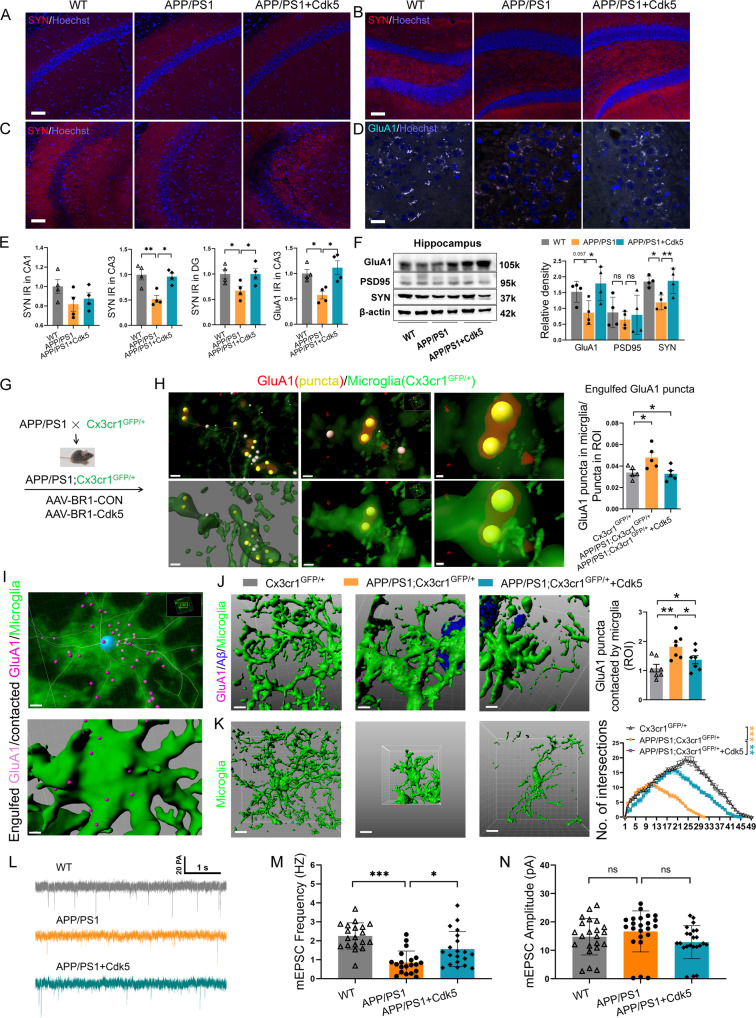
Wild-type serum rescues synaptic loss and microglial branching complexity in APP/PS1 mice. Representative confocal images (**A**–**D**) and quantification (**E**) of synaptophysin and GluA1 in the CA1, CA3, and DG in the hippocampus of WT mice, APP/PS1 mice treated with AAV-Cdk5, and the controls. IR: immunoreactivity; Scale bar, 100 μm. **F** Western blot analysis of synaptic proteins including GluA1, PSD95, and synaptophysin in the hippocampus. Data are represented as the mean ± s.e.m.; one-way ANOVA and LSD multiple comparison tests, *n* = 4 mice per group (more than 10 image fields from 5 brain slices per animal were analyzed and averaged as a value); *p < 0.05; **p < 0.01, ns nonsignificant. **G** Schematic of the experiments in **H**–**K**. **H** Confocal images and 3D reconstruction of microglial processes (Cx3cr1*-*GFP) engulfing GluA1^+^ puncta, the percentage of engulfed puncta by microglia was assessed. Data are represented as the mean ± s.e.m.; one-way ANOVA and LSD multiple comparison tests, *n* = 5 mice per group (more than 34 microglia from 5 brain slices per animal were analyzed and averaged as a value); *p < 0.05; **p < 0.01. **I** Engulfed puncta distributed inside microglia, the distance is less than 0 μm from the microglial surface; and contacted puncta distributed outside microglia, but the distance is less than 0.25 μm from the microglial surface. Purple puncta were shown in **I** (below). **J** 3D reconstruction of microglial processes and GluA1^+^ puncta in WT mice, as well as Aβ plaques (blue) in APP/PS1 mice, and contacted GluA1 puncta (less than 0.25 μm) were analyzed (*n* = 25–44 cells from 7 mice). **K** Sholl analysis of 3D-reconstructed microglia (*n* = 14–21 cells from 3 mice per group). *p < 0.05; **p < 0.01. Scale bar: 1 μm in **H**, **I**; 5 μm in **J**, 10 μm in **K**. **L** Representative traces of mEPSCs in DG pyramidal neurons from wide-type mice, APP/PS1 mice, and APP/PS1 mice treated with AAV-Cdk5, Scale bar: 1 s, 20 pA. **M** Average frequencies of mEPSCs. **N** Average amplitudes of mEPSCs; Kolmogorov–Smirnov test; *p < 0.05; ***p < 0.001; *n* = 19 or 20 cells from 3 mice/group in **M** and **N**.

Synaptic loss is usually detected early in AD patients [51] and in mouse models of the disease [52]. Thus, we next determined the effect of serum on the presynaptic marker synaptophysin and AMPA receptor GluA1. Consistent with previous studies [53, 54], the 8–9-month-old APP/PS1 male mouse model showed a significant decrease in synaptophysin and GluA1 expression in the DG and CA3, but not in CA1 of the hippocampus, compared to observations in the age-matched WT controls (Fig. 7A–E; for synaptophysin, decreases of 32.6% and 47.8% in the DG and CA3, respectively; decreases of 42.5% in CA3 for GluA1). After viral-induced Cdk5 overexpression, we found that the decreases in synaptic proteins were significantly restored in APP/PS1 mice (Fig. 7E, F), which is consistent with the results in APP/PS1 mice treated with serum and Ly6G antibody (Supplementary Fig. 13A–H). In addition, no differences in GluA1, synaptophysin or PSD95 expression in the cortex were observed in the different groups by western blot analysis (Supplementary Fig. 13I, J).

Microglia promote synapse pruning in a complement-dependent manner [52]. The synaptic loss observed in AD mice may be due to increased microglial phagocytosis [27, 52]. To test whether microglia directly interact with neuronal synapses in the present study, we investigated the association of microglia expressing fluorescent reporters with synaptic puncta (GluA1^+^) using 3D-reconstruction images of the hippocampus in APP/PS1 mice (Fig. 7G). We classified the GluA1^+^ synapses into two subsets-engulfed puncta (distributed inside microglia with a distance less than 0 μm from the microglial surface; yellow puncta in Fig. 7H; light purple puncta in Fig. 7I) and contacted puncta (distributed outside microglia with a distance less than 0.25 μm from the microglial surface; purple puncta in Fig. 7I, below). Our data revealed that significant decreases in GluA1^+^ synaptic spots engulfed or contacted by Cx3cr1^GFP^ microglia were rescued in Cdk5-overexpressed APP/PS1 mice (Fig. 7H–J). Sholl analysis showed that reduced microglial branching complexity in APP/PS1 mice was normalized by overexpression of Cdk5 in brain endothelial cells (Fig. 7K).

To further examine whether the impaired synaptic function in APP/PS1 mice was rescued after AAV-Cdk5 treatment, we examined the synaptic properties of pyramidal neurons in the DG. Whole-cell recordings from pyramidal neurons showed that the frequency, but not the amplitude of spontaneous mEPSC was dramatically decreased in APP/PS1 mice, whereas these effects were restored to wide-type levels after Cdk5 overexpression (Fig. 7L–N). Together with the data of GluA1^+^ synapse, these data indicated that Cdk5 overexpression restored the reduced synaptic inputs in DG pyramidal neurons in APP/PS1 mice.

As expected, microglia in APP/PS1 mice that received isotype IgG antibody displayed an amoeboid morphology, expressed high levels of CD68, and showed smaller volumes (Supplementary Fig. 14A–C). Notably, Cdk5 overexpression, serum and Ly6G antibody treatment not only significantly alleviated excessive activation of microglia (Iba-1^+^/CD68^+^), but also partially shifted the process length and volume of microglia toward the homeostatic form (Supplementary Fig. 14A–F). Serum neither altered the Aβ distributions (Supplementary Fig. 15A–D) nor RIPA and SDS-soluble Aβ_40_ and Aβ_42_ in the brain (Supplementary Fig. 15E–L), suggesting these effects on microglia are independent of Aβ plaques. In addition, we identified the levels of phosphor-Tau and total Tau in the brain after serum treatment, which had no influence on the total Tau and phosphor-Tau levels in both the hippocampus and cortex of APP/PS1 brain (Supplementary Fig. 16A–D). Furthermore, Tau multimers of 140 kDa (high molecular-weight band) were also measured after serum injection. Similarly, high molecular-weight Tau levels remained unchanged compared with the controls (Supplementary Fig. 16A–D). Altogether, our results revealed that inhibition of neutrophil infiltration by serum improved cognitive impairment, partially by alleviating synaptic loss and neuroinflammation in the hippocampus of the APP/PS1 brain.

## Discussion

The innate immune system including peripheral neutrophils and central microglia plays an important role in neurodegenerative disorders [4, 6, 39, 55]. Neutrophils are the key effector cells of early innate immunity and contribute to inflammation in the brain [10, 56] and periphery [6]. In this study, we found that serum injection blocked the recruitment of hyperreactive neutrophils into the brain and reduced neutrophil extravascular traps (NETs), thereby improving memory impairment in AD mice. Mechanistically, our study elucidated the trafficking mechanisms underlying neutrophil infiltration related to serum factor VEGF-A, which mediated endothelial Cdk5 activity in decreasing CXCL1 secretion in the AD brain. We further revealed that inhibition of neutrophil infiltration into the AD brain by serum and endothelial Cdk5 overexpression improved memory deficits, at least partially by reducing synapse pruning by microglia and elevating synaptic activity in the hippocampus.

### Wild-type serum injection may improve cognitive deficits by blocking neutrophil infiltration

Previous research showed that blocking Ly6G signaling, a neutrophil-specific marker, decreased neutrophil migration to vascular and neural inflammatory sites [57]. As a result, we demonstrated that serum injection dramatically reduced neutrophil adherence, particularly near brain vessels and Aβ plaque, and improved memory impairment in AD mice. Additionally, following serum injection, brain capillary segments were restored and neutrophil infiltration was blocked, increasing cerebral blood flow and perfusion [10], which improved cognitive abilities. The PPI analysis based on the DEPs also showed serum-derived cytokine VEGF-A and CXCL1-mediated chemotaxis. Consistently, recombinant VEGF-A had similar effects to Ly6G antibody in blocking neutrophil infiltration into the AD brain. We, therefore, concluded that VEGF-A in wild-type derived serum improved memory impairment in AD mice, most likely as a result of decreased neutrophil infiltration.

### VEGF-A may inhibit CXCL1 expression via endothelial Cdk5 activity

VEGF signaling is markedly reduced in the brain with aging while increasing VEGF signaling protects against age-related capillary loss, compromised perfusion, and reduced tissue oxygenation [22], which are also observed in AD patients [58–60]. Moreover, VEGF in CSF is associated with longitudinal memory performance, particularly in amyloid and Tau-positive individuals [61], and VEGF-related variants might appear to influence the risk for AD [62]. Therefore, these reports suggested that VEGF signaling is involved in the development of AD. Previous studies indicated that inhibition of Cdk5 activity in rat pituitary cells reduced VEGF-A expression [63, 64], implying that an underlying link exist between VEGF-A and Cdk5 in the endothelium. Interestingly, in the present study, recombinant VEGF-A directly prevented Aβ-mediated Cdk5 inhibition and promoted endothelial cell proliferation in bEnd.3 cells, and prevented brain capillary loss in an AD mouse model.

CDK5, a member of the cyclin-dependent kinase family, regulates endothelial cell proliferation, migration, and angiogenesis, and is involved in the major pathological features of AD [65–68]. In our study, endothelial Cdk5 overexpression inhibited CXCL1 secretion and peripheral neutrophil infiltration into the AD brain. Indeed, a prior study reported that endothelial-specific *Cdk5* knockout led to spontaneous seizures in mice by upregulating CXCL1-induced astrogliosis [48]. Together with the findings of lower *Cdk5* mRNA expression in the hippocampus [69] and increased *CXCL1* gene expression in prefrontal cortical microvessels of Tau-related AD patients [70], these results indicate that *Cdk5* deficiency in brain endothelial cells promotes CXCL1 secretion [71]. Thus, our study demonstrates that serum-derived VEGF-A suppress CXCL1 secretion by modulating Cdk5 activity and thereby preventing neutrophil migration into the brain.

### Infiltrating neutrophils may interact with microglia pruning by NET

The interaction of neutrophils and microglia has been reported in an LPS-induced inflammatory model using two-photon intravital microscopy [72]. Additionally, microglia-derived IL-1β and the chemokine CXCL1 recruit neutrophils to a fungus-infected CNS, in a Syk adaptor CARD9-dependent manner [45]. Here, we showed that microglial activation and synaptic loss were prevented by serum, endothelial Cdk5 overexpression, or blockade of neutrophil infiltration. Thus, neutrophils may provide proinflammatory signals to promote microglial pruning in an AD mouse model. Interestingly, we demonstrated that infiltrating neutrophils released NETs in several brain areas in an AD mouse model, which was prevented by anti-Ly6G antibody and serum treatment. This interaction may represent an intriguing mechanism for NET-mediated microglial activation and pruning of neuronal synapses [73]. Future studies are needed to explore the roles of neutrophil-producing NETs in microglial activation and synaptic pruning in an AD mouse model.

Importantly, we had previously demonstrated that the recruitment of monocytes to the brain via immune activation could exert beneficial effects against AD [27, 74, 75]. In the current study, although peripheral monocytes were increased after serum treatment, we found that neutrophil infiltration, rather than monocytes/macrophages were significantly decreased in the brain of APP/PS1 mice. This is the reason we specifically focus on the crucial roles of infiltrating neutrophils in AD pathologies. Blood rejuvenation could also modulate the expression of various genes involved in neuronal signaling pathways toward normal levels. For example, there are systemic factors in young plasma that are necessary for therapeutic benefits, including activation of Notch signaling depending on regeneration [76], restoration of growth differentiation factor 11 pathway [12, 77], upregulation of oxytocin levels [78] and activation of the cyclic AMP response element binding protein.

### Wild-type serum-derived VEGF-A may exert a beneficial effect on an AD mouse model

VEGF-A is a neuroprotective cytokine that promotes neurogenesis and angiogenesis in the brain [79, 80]. Consistently, we provided evidence that serum rescued the insufficient circulating VEGF-A level in AD mice. Recombinant VEGF-A promoted endothelial cell proliferation in vitro and prevented the capillary loss in vivo. Thus, VEGF-A may be important to improve cerebral perfusion and blood flow for sufficient blood oxygen, especially in age [22, 79] and Alzheimer’s disease [81]. Therefore, endothelial VEGF-A/Cdk5 signaling may be a potential therapeutic strategy to improve AD pathology through regulating neutrophil infiltration.

In addition to VEGF-A, other enriched blood-borne factors, such as VCAM1, HGF, and clusterin, which are involved in vascular endothelial cell function, may also improve brain functions. Indeed, a brain endothelial *VCAM1* gene deficiency counteracts the detrimental effects of aged plasma on young brains, including microglial reactivity and memory function [16]. HGF has recently been shown to have beneficial effects on hippocampal neurogenesis and cognitive function in SAMP8 mice, another mouse model of AD [82]. Clusterin, a complement cascade inhibitor, can bind to brain endothelial cells and reduce neuroinflammation in mThy-1-hAPP751 mice [83]. Whether other serum factors work together with VEGF-A is still unknown, but our work extends the application of blood-borne factors to restore AD brain functions. More targeted or combined therapy using serum factors warrants further studies.

### How VEGF-A injection affects BBB permeability in an AD mouse model

VEGF has broad functions in multiple signaling pathways in a variety of cell types. For example, blocking the effect of VEGF on astrocytes reversed BBB breakdown [84], while inhibiting luminal VEGF signaling increased BBB integrity and cerebral blood flow and reduced capillary density in APP/PS1 mice [85]. In this study, we observed that a low-dose VEGF-A injection restored brain capillary density in the same strain of the AD mouse model, which is consistent with the data showing that anti-VEGF treatment reduced capillary density in Ali’s work. However, no significant changes in BBB integrity and permeability were observed in our AD mouse model after the VEGF-A injection. The possible reason is that the APP/PS1 mouse model (8 months) used by us is younger than the animals (10–14 months) used in Ali’s study. An alternative explanation is that a transient increase in BBB permeability may occur after low-dose VEGF-A treatment, which can be restored within several hours [86]. Therefore, understandably, BBB permeability and integrity were not further worsened in VEGF-A-treated APP/PS1 mice.

Moreover, many circulating proteins have been identified to be involved in the regulation of BBB permeability, such as endothelin-1 (EDN1) and fibrinogen (FG), which were reported to be increased in the frontal cortex of AD patients [87]. Therefore, the particular role of VEGF-A in BBB permeability, capillary density and blood flow depending on cell types, disease models and disease stages must be elucidated. Additionally, the question of whether 8-month-old serum could have the same positive effects on AD as 4–6-month-old serum still remained unsolved. It is a limitation in the present study that needs to be addressed in the future.

## Conclusion

**Fig. 8:**
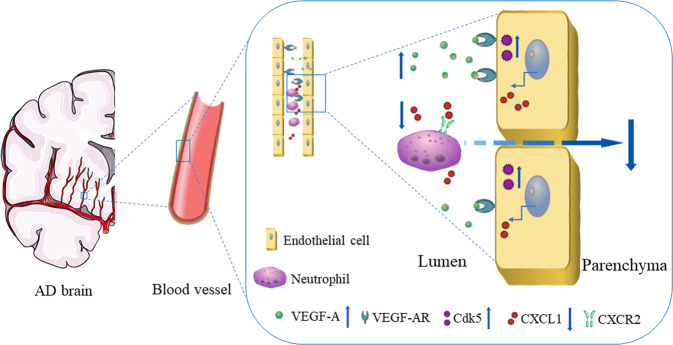
Graphical abstract of this study. The proposed role of endothelial VEGF-A/Cdk5/CXCL1 signaling in the process of neutrophil infiltration into the brain in AD mice. Wild-type serum-derived VEGF-A suppresses brain vascular endothelial CXCL1 expression via up-regulation of Cdk5 activity, thereby stopping neutrophils recruitment into the brain in AD mice, and improving memory impairment. VEGF-AR VEGF-A receptor, CXCR2 C-X-C motif chemokine receptor 2.

Blood-borne factors from young or exercise serum/plasma have been proposed to benefit brain functions, including neurogenesis, synaptic plasticity, and inflammation as well as learning and memory, both in aged animals and in AD mouse models. Thus, the application of blood factors in other neurodegenerative diseases warrants consideration. Our data provide evidence that endothelial VEGF-A/Cdk5 signaling mediates neutrophil-trafficking molecules, highlighting that brain endothelial VEGF-A/Cdk5 may serve as a potential therapeutic target for Alzheimer’s disease (Fig. 8).

### Supplementary information

Supplementary Table 1

Supplementary Table 2

Supplementary Table 3

Supplementary materials

Figure S1

Figure S2

Figure S3

Figure S4

Figure S5

Figure S6

Figure S7

Figure S8

Figure S9

Figure S10

Figure S11

Figure S12

Figure S13

Figure S14

Figure S15

Figure S16
