# Supplementary material for: VEGF-A in serum protects against memory impairment in APP/PS1 transgenic mice by blocking neutrophil infiltration
Source: Mol Psychiatry. 2023 Jun 6;28(10):4374–89. doi: 10.1038/s41380-023-02097-w (PMC10827659; doi:10.1038/s41380-023-02097-w)
Supplement: Supplementary file 3 — Supplementary Table 3 [file 41380_2023_2097_MOESM3_ESM.docx]

**Table 3 Hematologic profile of wide-type mice, APP/PS1 mice, and APP/PS1 mice treated with wild-type serum**

| Parameters | Wide-type  (*n* = 15) |  | APP/PS1  (*n* = 12) | APP/PS1+serum  (*n* = 5) | *F* | *P* value  (APP/PS1 *vs.* APP/PS1+serum) |
| --- | --- | --- | --- | --- | --- | --- |
| WBC/10^9^/L | 5.58 ± 2.77 |  | 13.6 ± 5.00 | 9.06 ± 2.26 | 15.437 | 0.062^2)^ |
| RBC/10^9^/L | 8.13 ± 1.22 |  | 8.09 ± 1.05 | 6.41± 0.76 | 5.020 | 0.008 |
| Neu/10^9^/L  Lym/10^9^/L  Mon/10^9^/L  Eos/10^9^/L  Bas/10^9^/L  HGB (g/L)  HCT/%  MCV/fL  MCH/pg  MCHC (g/L) PLT/10^9^/L  MPV/fL PDW | 1.09 ± 0.43  4.10 ± 2.46  0.19 ± 0.16  0.19 ± 0.10  0.01 ± 0.01  131.67 ± 18.46  37.77 ± 5.27  46.57 ± 1.69  16.24 ± 0.74  348.67 ± 12.60  944.93 ± 375.42  5.41 ± 0.41  15.41 ± 0.26 |  | 4.05 ± 1.20  8.82 ± 4.28  0.43 ± 0.28  0.21 ± 0.10  0.09 ± 0.05  131.58 ± 17.89  37.42 ± 4.90  46.26 ± 1.74  16.23 ± 0.60  351.08 ± 6.61  892.25 ± 307.85  6.51 ± 0.44  15.84 ± 0.41 | 2.87 ± 1.01  5.17 ± 1.51  0.76 ± 0.41  0.23 ± 0.05  0.02 ± 0.02  106.40 ± 10.38  30.48 ± 2.25  47.74 ± 2.68  16.66 ± 0.69  349.00 ± 8.63  1182.60 ± 205.54  6.52 ± 0.65  16.10 ± 0.49 | 38.331  7.514  11.228  0.445  18.530  4.465  4.463  1.115  0.843  0.199  1.359  22.727  8.936 | 0.017*^,1)^  0.060^2)^  0.014*^,1)^  0.681  0.009**^,2)^  0.011*  0.011*  0.149  0.249  0.703  0.111  0.962  0.709^2)^ |

**P<*0.05. The data are shown as the mean ± SD from three groups. The statistically significant differences were tested for one-way ANOVA and Dunnett T3 when the homogeneity of variance is equal ^1)^ or unequal ^2)^, respectively. This is a supplementary table.
