# Supplementary material for: VEGF-A in serum protects against memory impairment in APP/PS1 transgenic mice by blocking neutrophil infiltration
Source: Mol Psychiatry. 2023 Jun 6;28(10):4374–89. doi: 10.1038/s41380-023-02097-w (PMC10827659; doi:10.1038/s41380-023-02097-w)
Supplement: Supplementary file 4 — Supplementary materials [file 41380_2023_2097_MOESM4_ESM.docx]

**Supplementary figure and table legends**

**Fig. S1 Wild-type serum injection modulates peripheral and brain monocytes in APP/PS1 mice**

Flow cytometry sorting and gating strategy for immune cells (P1), single cells (P2), CD45^+^ leukocytes (P3), and CD45^hi^CD11b^+^ monocytes (P4) in the PBMCs (A-C) and the brain (D-F) of APP/PS1 mice treated with serum and PBS. (C and F) Flow cytometry analysis of the frequencies of CD45^hi^CD11b^+^ monocytes in the periphery (C) and the brain (F). The arrows in A and D represent the P2 subset based on P1, the P3 subset based on P2, and the P4 subset based on P3. All data are shown as the mean±s.e.m.; unpaired Student’s two-tailed *t-*test, **p* < 0.05, *n* = 6 per group.

**Fig. S2 KEGG pathway enrichment analysis of differentially expressed cytokines in the periphery**

(A-B) KEGG enrichment analysis of 23 downregulated cytokines (A) and 59 upregulated cytokines (B) in the serum from APP/PS1+Serum *vs.* APP/PS1+PBS mice. Cytokine-cytokine receptor interactions and the chemokine signaling pathway are enriched as indicated by the red box in A and B.

**Fig. S3 Cerebral cytokine levels are measured in the brain of WT+PBS, APP/PS1+PBS, and APP/PS1+Serum mice**

1. C, F, and I) The levels of CXCL1 (A), CCL3 (B), CXCL16 (C), FetuinA (F), and IL-22 (I) were significantly increased in the brains of APP/PS1 mice compared to those of age-matched WT mice. However, these increases were restored to the levels detected in WT+PBS mice after serum treatment. (D, G, H, and J) No obvious differences in the levels of CXCL9 (D), EGF (G), FGF2 (H), and TGFB1 (J) were detected in the brains of APP/PS1+PBS and APP/PS1+Serum mice. Mean concentrations are expressed in pg/mL of hippocampal homogenates. All data are shown as the mean±s.e.m.; one-way ANOVA followed by Bonferroni multiple comparison tests; *n* = 6 mice per group; **p* < 0.05, ***p* < 0.01, and ****p* < 0.001.

**Fig. S4 Correlation analysis between CXCL1 levels and Cdk5 and pCdk5 protein levels in bEnd.3 cells**

(A-B) Supernatant CXCL1 was negatively correlated with Cdk5 levels in A (Pearson’s *r* = -0.667, *p* < 0.001) and pCdk5 levels in B (Pearson’s *r* = -0.707, *p* < 0.0001). The data points are shown with 95% CIs and linear regression. (C-D) Representative confocal images of Cdk5 and pCdk5 colocalizing with CXCL1 in bEnd.3 cells (mouse brain microvascular endothelial cells, MBECs) are shown in (C) and (D). Scale bars: 20 μm in C, 5 μm in D. (E-F) Analysis of the colocalization of Cdk5 and pCdk5 with CXCL1. Fluorescence intensity profiles of Cdk5 (E) and pCdk5 (F) with CXCL1 show negative expression according to the fluorescence signal across the red arrow in C and D, respectively.

**Fig. S5 Wild-type serum treatment increases peripheral VEGF-A levels of APP/PS1 mice**

Peripheral VEGF-A levels from 4-week-old, and 8-week-old WT mice, WT mice (8 months old), and APP/PS1 mice (8 months old) treated with serum or not were measured by ELISA kit. VEGF-A levels were significantly decreased in the 8-month-old APP/PS1 mice compared to the age-matched WT mice; whereas these effects were reversed after serum treatment. All data are shown as the mean±s.e.m.; one-way ANOVA and followed by Bonferroni multiple comparison tests; *n* = 14 mice per group; ns: nonsignificant; **p* < 0.05; ***p* < 0.01.

**Fig. S6 Inhibition of Cdk5 activity prevents the effect of VEGF-A on CXCL1 expression in bEnd.3 cells**

1. Flow cytometry analysis of CD31/VEGFR2-positive brain vessel endothelial cells in bEnd.3 cells. Almost all cells are CD31 and VEGFR2 double positive. (B-F) Flow cytometry analysis of the frequencies of CXCL1-positive cells in CD31/VEGFR2-positive endothelial cells treated with Aβ (2 μM), recombinant VEGF-A protein (20 ng/mL), and roscovitine (20 μM). (G) Quantitative analysis of VEGF-A levels related to B-E in the supernatant by ELISA kit. One-way ANOVA followed by Bonferroni multiple comparison tests. ns: nonsignificant; *n* = 4 per group; ns: nonsignificant; **p* < 0.05; ***p* < 0.01; ****p* < 0.001.

**Fig. S7 Wild-type serum injection and recombinant VEGF-A promote Cdk5 and pCdk5 activities of APP/PS1 mice**

(A-P) Representative confocal images of Cdk5 (A-E) and pCdk5 (I-M) in the brain slices of WT and APP/PS1 mice that were treated with or without serum, Ly6G antibody, and VEGF-A. (F and N) Quantification of Cdk5 (F) and pCdk5 (N) expression (red) in the brain vessels (lectin, purple) in the hippocampus of indicated groups after behavioral tests, as indicated by the inserted boxes. Higher magnification (zoom 2 at 63 × magnification) images as indicated by the white boxes are shown at G-H and O-P. All data are shown as the mean±s.e.m.; significances were calculated using one-way ANOVA followed by Bonferroni multiple comparison tests. *n* = 4-5 mice per group; ****p* < 0.001. Scale bar: 100 μm in A-E; I-M; 20 μm in high-magnification images in G-H and O-P.

**Fig. S8 VEGF-A injection does not affect BBB permeability or Claudin 5 expression in APP/PS1 mice**

(A-B) Representative confocal images in the cortical areas from different groups (A), revealed decreased claudin expression in APP/PS1 mice as compared to WT control mice (B). No significant differences were observed in claudin levels between VEGF-A-treated APP/PS1 mice and Isotype IgG-treated APP/PS1 mice. Scale bar: 20 μm in A. (C) Normalized absorbance of brain tissue, including cortex and hippocampus homogenates at 620 nm, to quantify the extravasation of Evans Blue leakage into the brain for VEGA-A or Isotype IgG-treated APP/PS1 mice and WT controls. All data are shown as the mean±s.e.m.; unpaired Student’s two-tailed *t* test for WT+Isotype *vs.* WT+VEGF-A or APP/PS1+Isotype *vs.* APP/PS1+VEGF-A groups. *n* = 3 mice per group; ns: nonsignificant; **p* < 0.05.

**Fig. S9 Neutrophil depletion improves learning and memory abilities, similar to the beneficial effects of serum treatment**

(A) All groups exhibited similar baseline freezing times during training. (B-H) Learning and memory were assessed in WT mice (8 months old) and APP/PS1 mice (8 months old) treated intravenously with serum derived from 4-to 6-week-old animals (200 µL, 9 injections) or not using fear conditioning and MWM paradigms. *n*=10 per group. The percentage of freezing time (%) was analyzed among different groups during contextual (B) or cued (C) fear conditioning testing. (D) Escape latencies of APP/PS1 mice injected with the serum, APP/PS1 mice subjected to neutrophil depletion with anti-Ly6G antibodies, age-matched WT mice, or AD model mice in the MWM test. *n*=10 per group. (E-F) Time (E) and distance (F) spent in the target quadrant (TQ) for the above groups. The frequency of platform crosses in the TQ (G) and the swimming velocity (H) during the spatial probe phase. All data are shown as the mean±s.e.m.; statistical analysis was performed using two-way ANOVA for the MWM test and Bonferroni multiple comparison tests; *n*=10 mice per group; **p* < 0.05; ^#^*p* < 0.05; ***p* < 0.01.

**Fig. S10 Neutrophil depletion is observed in the dura mater and brain with an anti-Ly6G neutralizing antibody**

1. B) Representative confocal images of neutrophils (Ly6G^+^, red cells) in the superior sagittal sinus (SSS) and non-SSS of APP/PS1 mice treated with anti-Ly6G antibodies (right panel) and isotype IgG antibodies (left panel). (C-D) Representative confocal images of neutrophils (Ly6G^+^ red cells in C) in the 3V around hippocampus and CP (Ly6G^+^ red cells in D) of APP/PS1 mice treated with anti-Ly6G antibodies (right panel) and isotype IgG antibodies (left panel). CP: choroid plexus; 3V: the third ventricle. (E-F) Quantification of Ly6G^+^ neutrophil counts in the dura mater and the brain in the APP/PS1+IgG mice and APP/PS1+anti-Ly6G antibody mice. All data are shown as the mean±s.e.m.; unpaired Student’s two-tailed *t* test, *n* = 4-5 mice per group; ****p* < 0.001. Scale bar: 50 μm in A-D.

**Fig. S11 Effects of Ly6G antibody on exploratory behavior after neutrophil depletion in the periphery**

Flow cytometry sorting and gating strategy for immune cells (P1), single cells (P2), and CD45^+^ leukocytes (P3) in the PBMCs (A-B; F-G) of APP/PS1 mice treated with serum and PBS. (C) Monocytes have distinctly different populations based on Ly6C expression. (D-E) Flow cytometry analysis of Ly6G^+^ neutrophil (indicated by the red gate in C), and Ly6C^+^ monocyte (indicated by the blue gate in C) frequencies of CD45^+^ cells in the PBMCs of isotype control antibody- and Ly6G antibody-treated APP/PS1 mice. The arrows in A and F represent the CD45^+^ cell subset (box) based on the single cells (box); the single cell subset is based on the cells in the elliptic box. (H-I) Flow cytometry analysis of CD3 lymphocytes (indicated by the red gate in H) frequency of CD45^+^ cells in the PBMCs of isotype control antibody- and Ly6G antibody-treated APP/PS1 mice. (J-L) Exploratory activities were assessed in APP/PS1 mice after neutrophil depletion by Ly6G antibody, as reflected by total distance (J), duration in the center area (K), and velocity of movement (L) in the OFT. All data are shown as the mean±s.e.m.; unpaired Student’s two-tailed *t*-test and one-way ANOVA and Bonferroni multiple comparison tests; *n*=4-5 mice per group for flow cytometry analysis; *n*=6 mice per group for the behavioral test; ns: nonsignificant; ns: nonsignificant; ****p* < 0.001.

**Fig. S12 Cdk5 expression is increased in the brains of BR1-mCdk5-treated APP/PS1 mice**

(A) Confocal micrographs of double immunostaining for lectin (purple) and Cdk5 (red) in the hippocampus of APP/PS1 mice after 2 weeks of BR1-mCdk5 treatment (i.v. injection). EGFP is considered the reporter element of rAAV2-BR1-CMV-mCdk5-P2A-EGFP. Hoechst staining is shown in blue. Scale bar: 100 μm. (B) Higher magnification of the boxed area in A. Scale bar: 20 μm in B.

**Fig. S13 Partial protection against hippocampal synaptic loss by wild-type serum treatment**

(A-D) A presynaptic marker (synaptophysin) and an AMPA receptor GluA1 were assayed by confocal microscopy (20 × magnification) in WT mice, APP/PS1 mice treated with serum, and Ly6G antibody. Synaptophysin staining in the CA1 (A), CA3 (B), and DG (C) of the hippocampus. (D) GluA1 staining in the hippocampus. (E-H) Quantification of synaptophysin and GluA1 immunoreactivity (IR) in the different regions of the hippocampus of AD mice. (I-J) Western blot analysis of synaptic proteins including GluA1, PSD95, and synaptophysin in the cortex of different groups. All data are shown as the mean±s.e.m.; one-way ANOVA and Bonferroni multiple comparison tests; *n* = 3-4 per group; **p* < 0.05; ***p* < 0.01; ****p* < 0.001. Scale bar, 100 μm in A-D.

**Fig. S14 Wild-type serum injection normalized the aberrant reactivity and morphological characteristics of microglia in the AD mouse model**

(A) CD68 and Iba-1 coimmunostaining in the DG zone of WT mice and APP/PS1 mice treated with serum, anti-Ly6G antibody, and AAV-Cdk5 delivery. (B-C) Quantification of the percentage of Iba-1 signaling and the percentage of CD68 signaling within Iba-1^+^ microglia. *n* = 5 mice per group. (D) 3D reconstruction of confocal microglia images indicated by the Cx3cr1-GFP signal (green) from APP/PS1 mice crossed with Cx3cr1-GFP mice (APP/PS1;Cx3cr1-GFP mice). (E-F) Quantifications of microglial dendrite length (E) and volume (F) in different groups. All data are shown as the mean±s.e.m.; One-way ANOVA followed by the Bonferroni *post hoc* test. *n* = 12 cells from 3 mice per group; **p* < 0.05; ***p* < 0.01; ****p* < 0.001 Scale bar: 50 μm in A; 3 μm in D.

**Fig. S15 Effects of wild-type serum on Aβ plaque counts and RIPA- and SDS-soluble Aβ levels in the brains of APP/PS1 mice**

(A-D) The numbers of small (<20 µm) and medium (20 to 50 µm) plaques were not changed in the cortex and the hippocampus of APP/PS1 mice after serum treatment. (E-K) Serum treatment did not affect RIPA- and SDS-soluble Aβ40 or Aβ42 levels in either the cortex or hippocampus of APP/PS1 mice. All data are presented as the mean ± s.e.m.; unpaired Student’s two-tailed *t*-test; *n* = 6-8 mice per group.

**Fig. S16 Effects of wild-type serum on Tau phosphorylation levels in the brains of APP/PS1 mice**

(A-B) Western blot for total Tau and phosphor-Tau Ser^199/202^, including Tau multimers in the hippocampus (A) and cortex (B) in WT mice and APP/PS1 mice APP/PS1 mice after serum treatment or not. (C-D) Serum injection did not cause changes in total Tau and phosphor-Tau Ser^199/202^ in the hippocampus (C) and cortex (D) of APP/PS1 mice. All data are presented as the mean ± s.e.m.; One-way ANOVA followed by the LSD *post hoc* test; *n* = 3 mice per group ns: nonsignificant; **p* < 0.05.

**Supplementary Table 1 The top 82 ranked proteins in wide-type and APP/PS1 mice treated with wild-type serum or not**

DEPs of the serum of WT+PBS, WT+Serum, APP/PS1+PBS, and APP/PS1+Serum mice using a mouse cytokine array in Sheet 1 of Table 1; DEPs, differently expressed proteins; Up, up-regulation, APP/PS1+Serum group *vs.* APP/PS1+PBS group; Down, down-regulation, APP/PS1+Serum group *vs.* APP/PS1+PBS group. Gene Ontology (GO) analysis using the DEPs was shown in Sheet 2 and Sheet 3 of Supplementary Table 1. This is a supplementary table.

**Supplementary Table 2 The 36 common proteins related to Figure 3E**

The 36 differently expressed proteins (DEPs) in the serum of WT+PBS, WT+Serum, APP/PS1+PBS, and APP/PS1+Serum mice were shown, including protein Uniprot and gene ID in Table 2. This is a supplementary table.

**Supplementary Table 3 Hematologic profile of wide-type mice, APP/PS1 mice, and APP/PS1 mice treated with wild-type serum**

**P*<0.05. The data are shown as the mean ± SD from three groups. The statistically significant differences were tested for one-way ANOVA and Dunnett T3 when the homogeneity of variance is equal ^1)^ or unequal ^2)^, respectively. This is a supplementary table.
