# Supplementary figures and images for: VEGF-A in serum protects against memory impairment in APP/PS1 transgenic mice by blocking neutrophil infiltration
Source: Mol Psychiatry. 2023 Jun 6;28(10):4374–89. doi: 10.1038/s41380-023-02097-w (PMC10827659; doi:10.1038/s41380-023-02097-w)

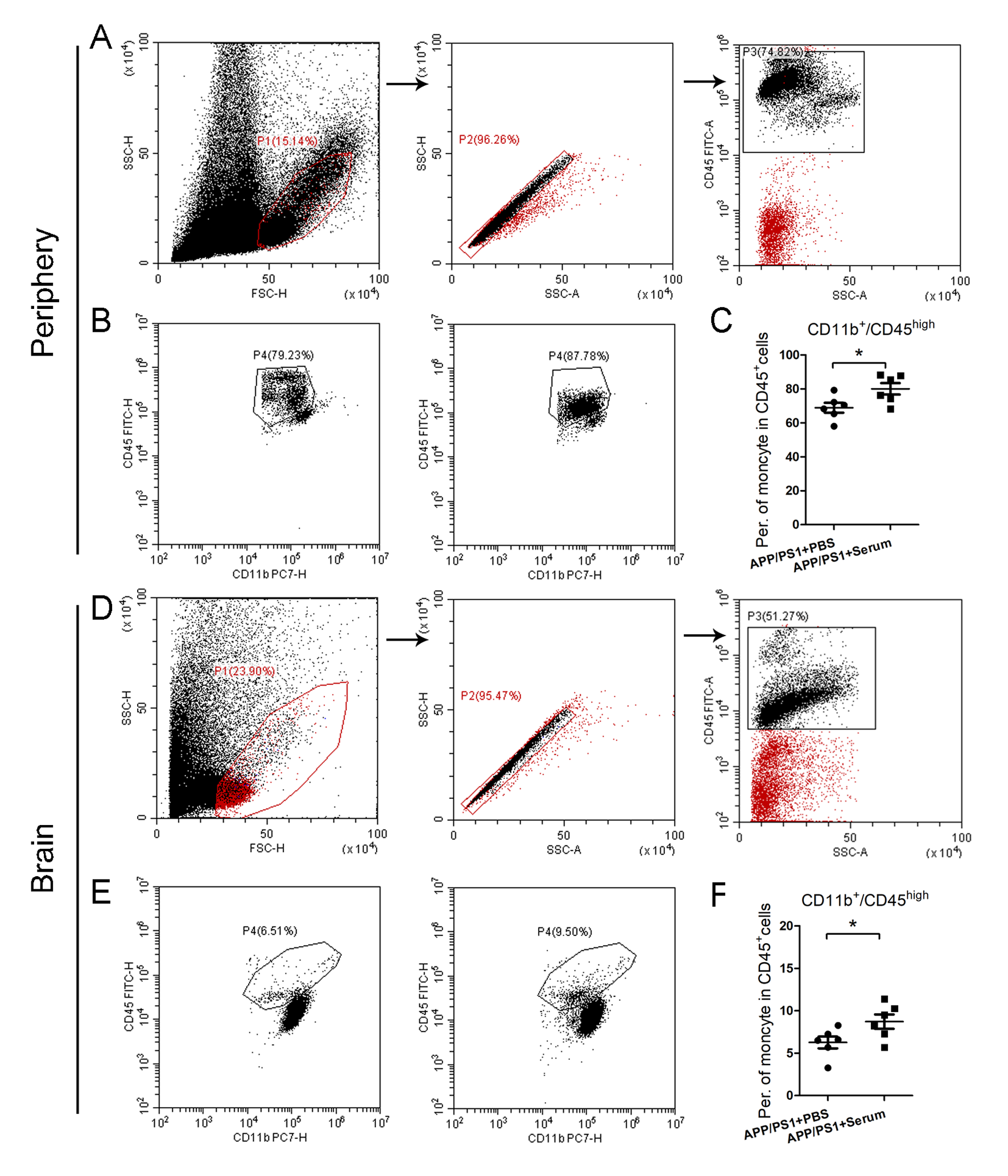

Supplement: Supplementary file 5 — Figure S1 [file 41380_2023_2097_MOESM5_ESM.tif]

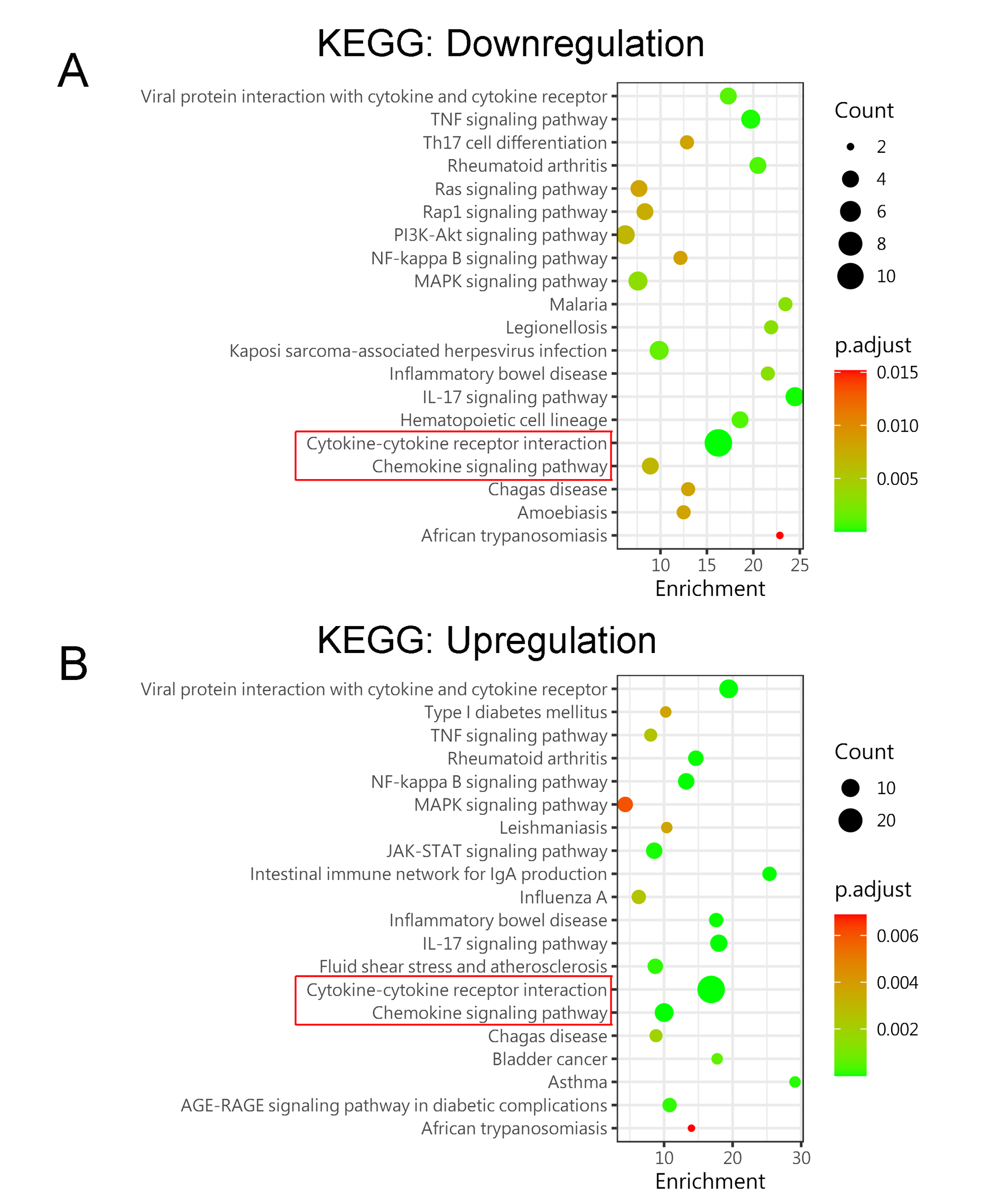

Supplement: Supplementary file 6 — Figure S2 [file 41380_2023_2097_MOESM6_ESM.tif]

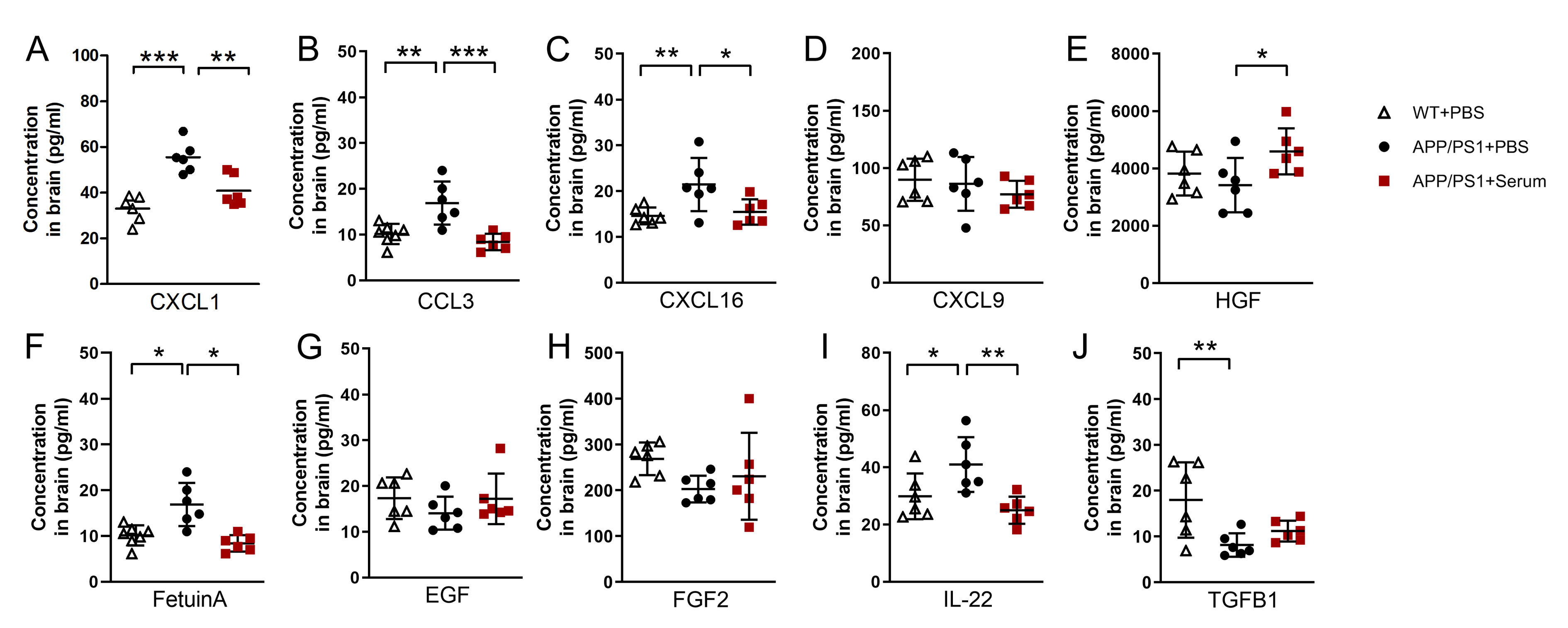

Supplement: Supplementary file 7 — Figure S3 [file 41380_2023_2097_MOESM7_ESM.tif]

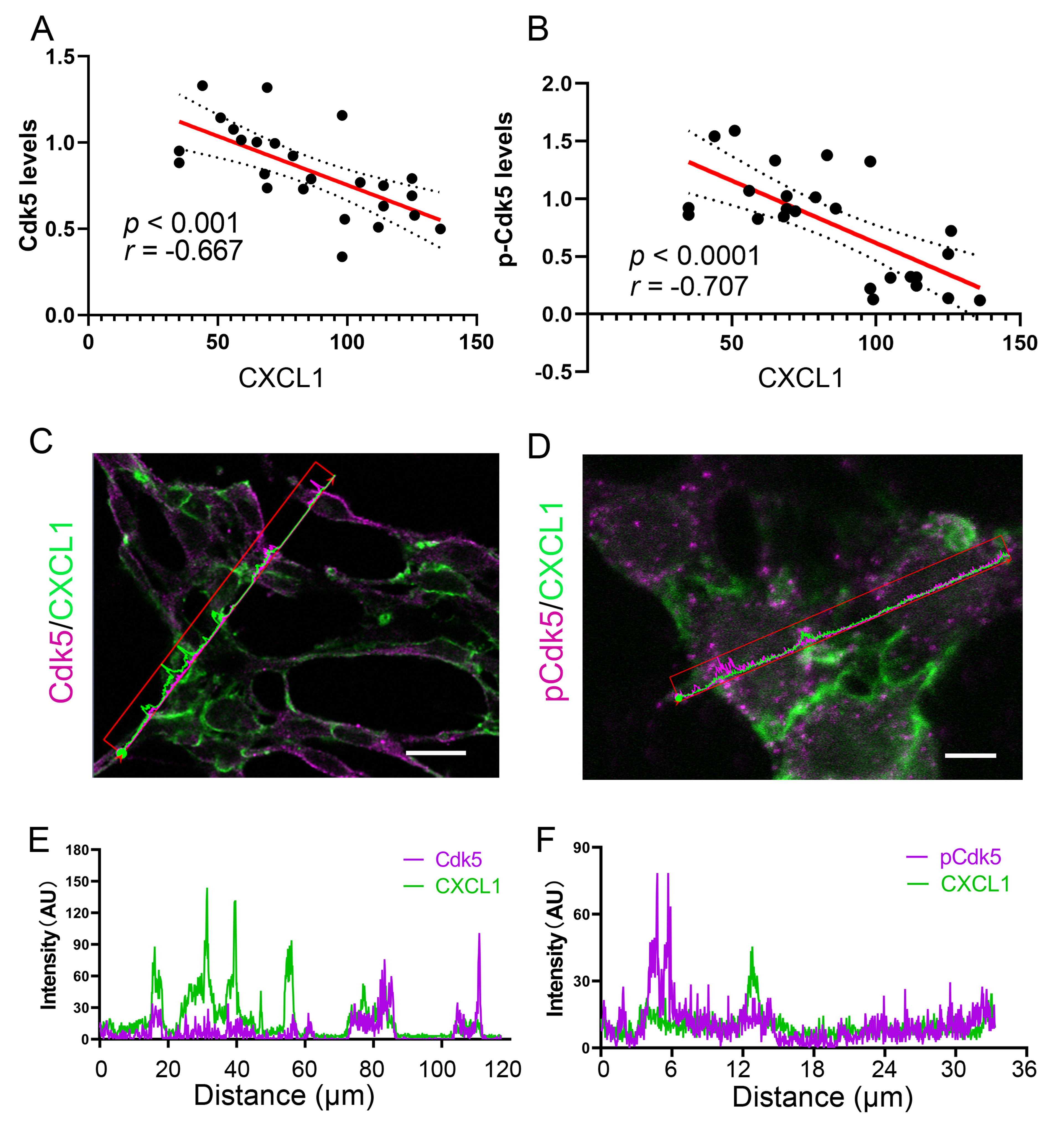

Supplement: Supplementary file 8 — Figure S4 [file 41380_2023_2097_MOESM8_ESM.tif]

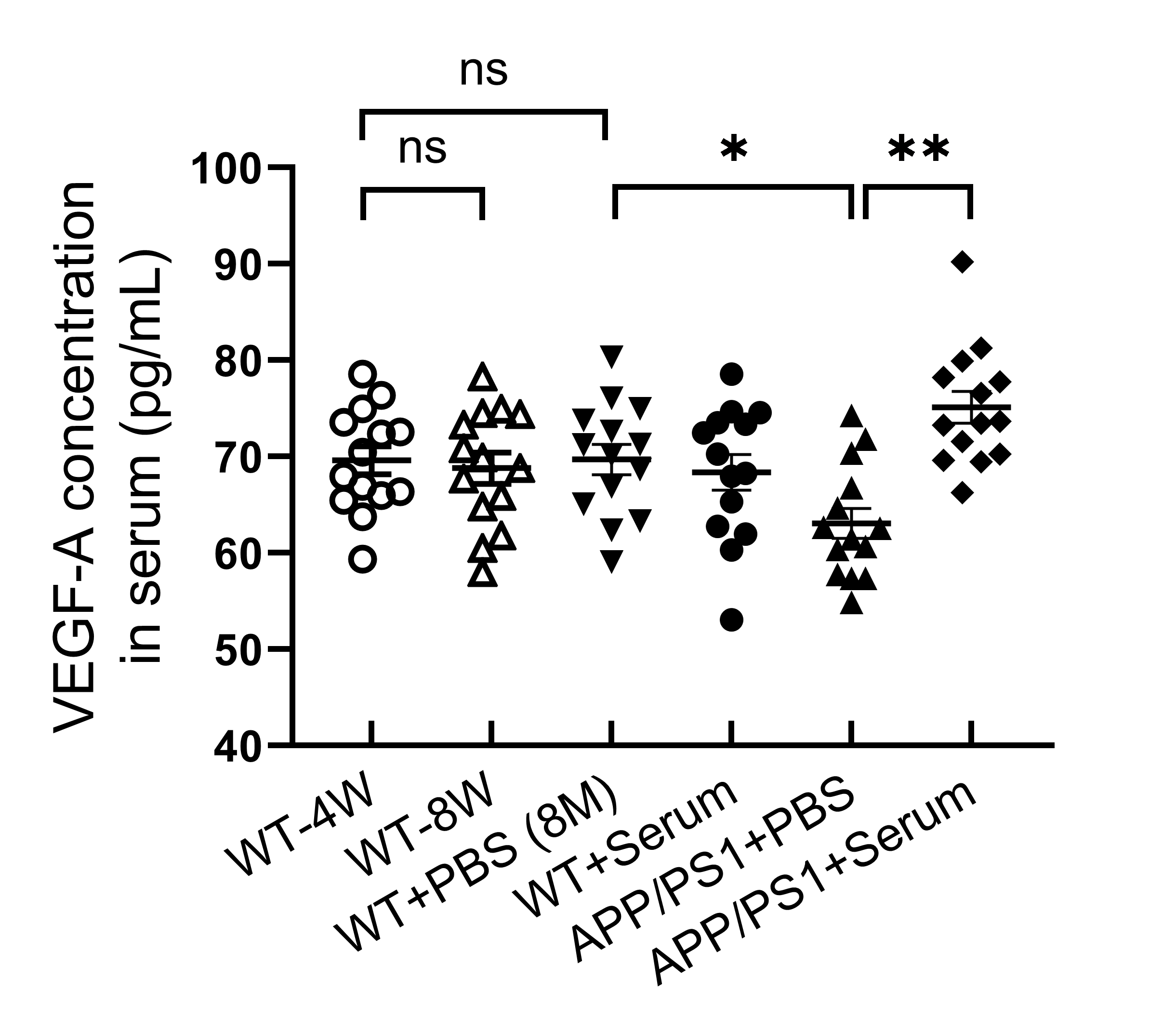

Supplement: Supplementary file 9 — Figure S5 [file 41380_2023_2097_MOESM9_ESM.tif]

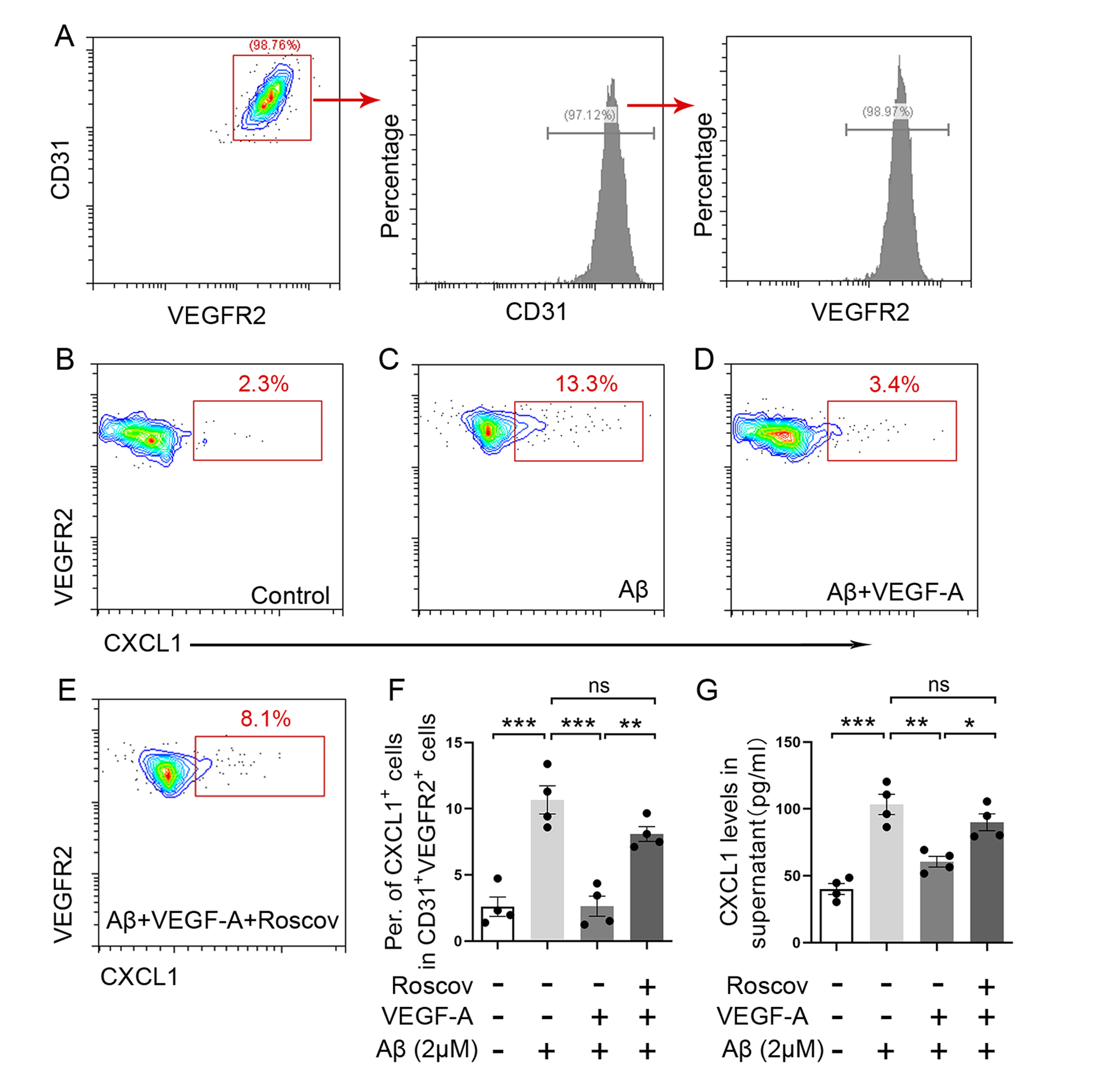

Supplement: Supplementary file 10 — Figure S6 [file 41380_2023_2097_MOESM10_ESM.tif]

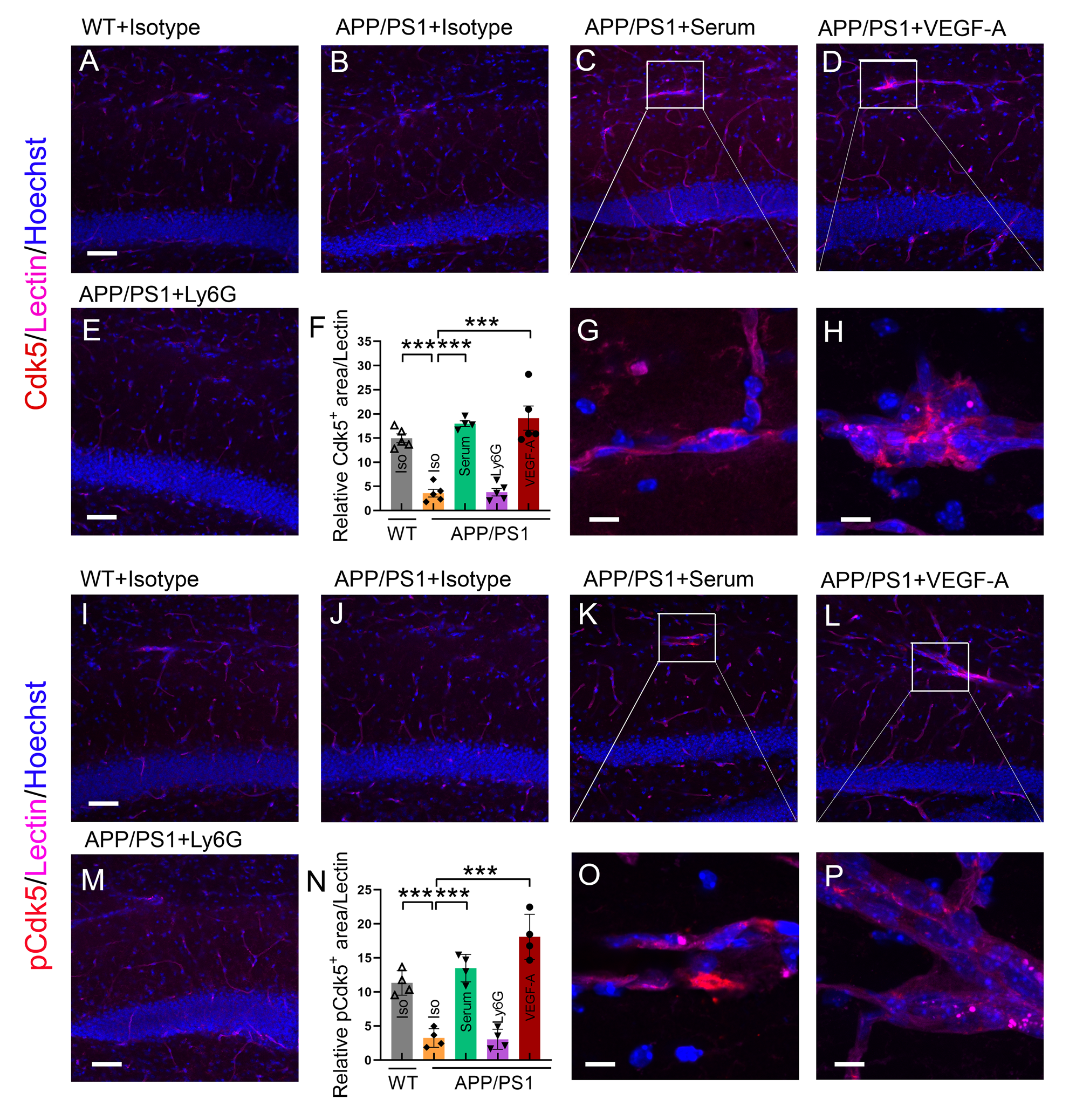

Supplement: Supplementary file 11 — Figure S7 [file 41380_2023_2097_MOESM11_ESM.tif]

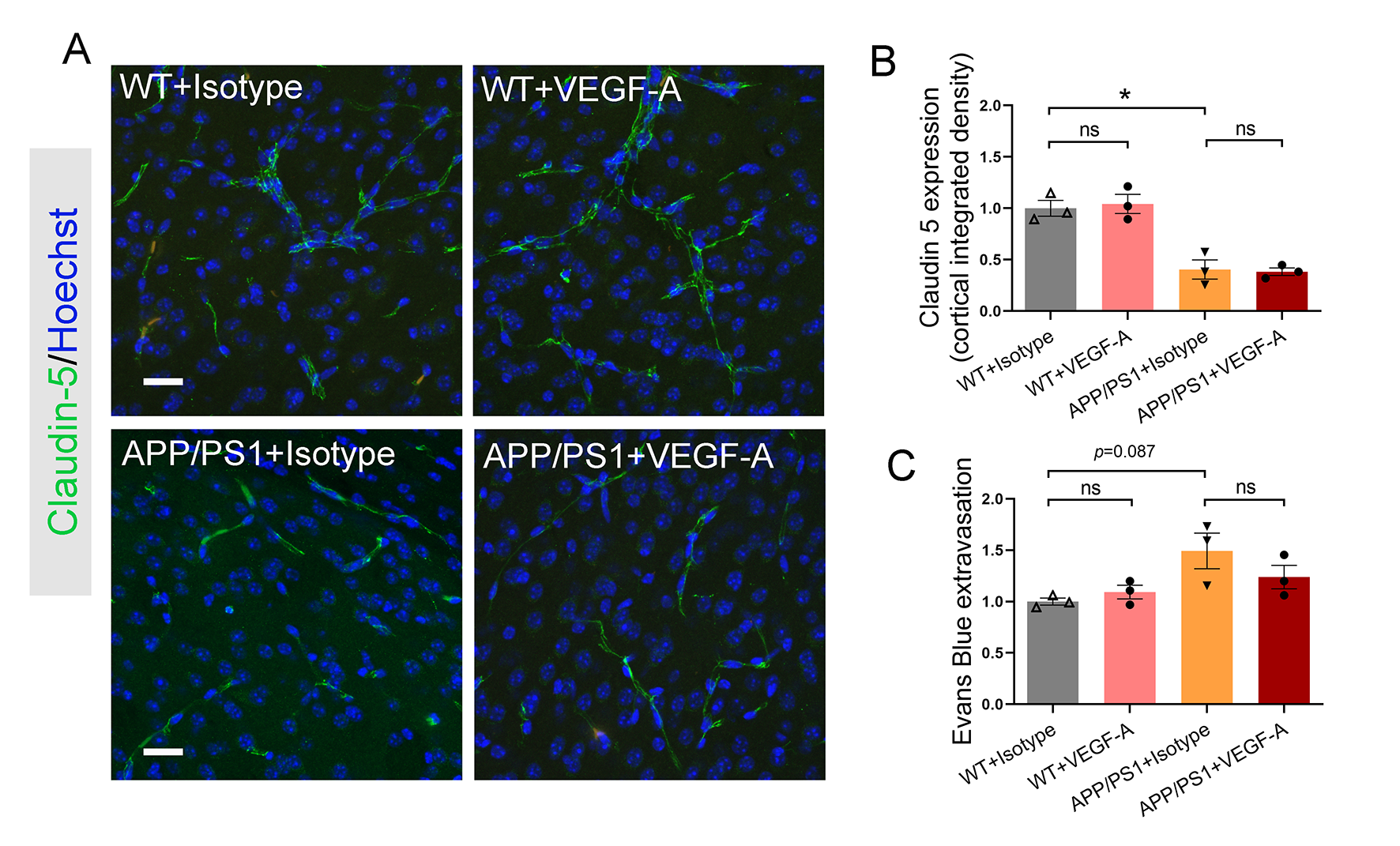

Supplement: Supplementary file 12 — Figure S8 [file 41380_2023_2097_MOESM12_ESM.tif]

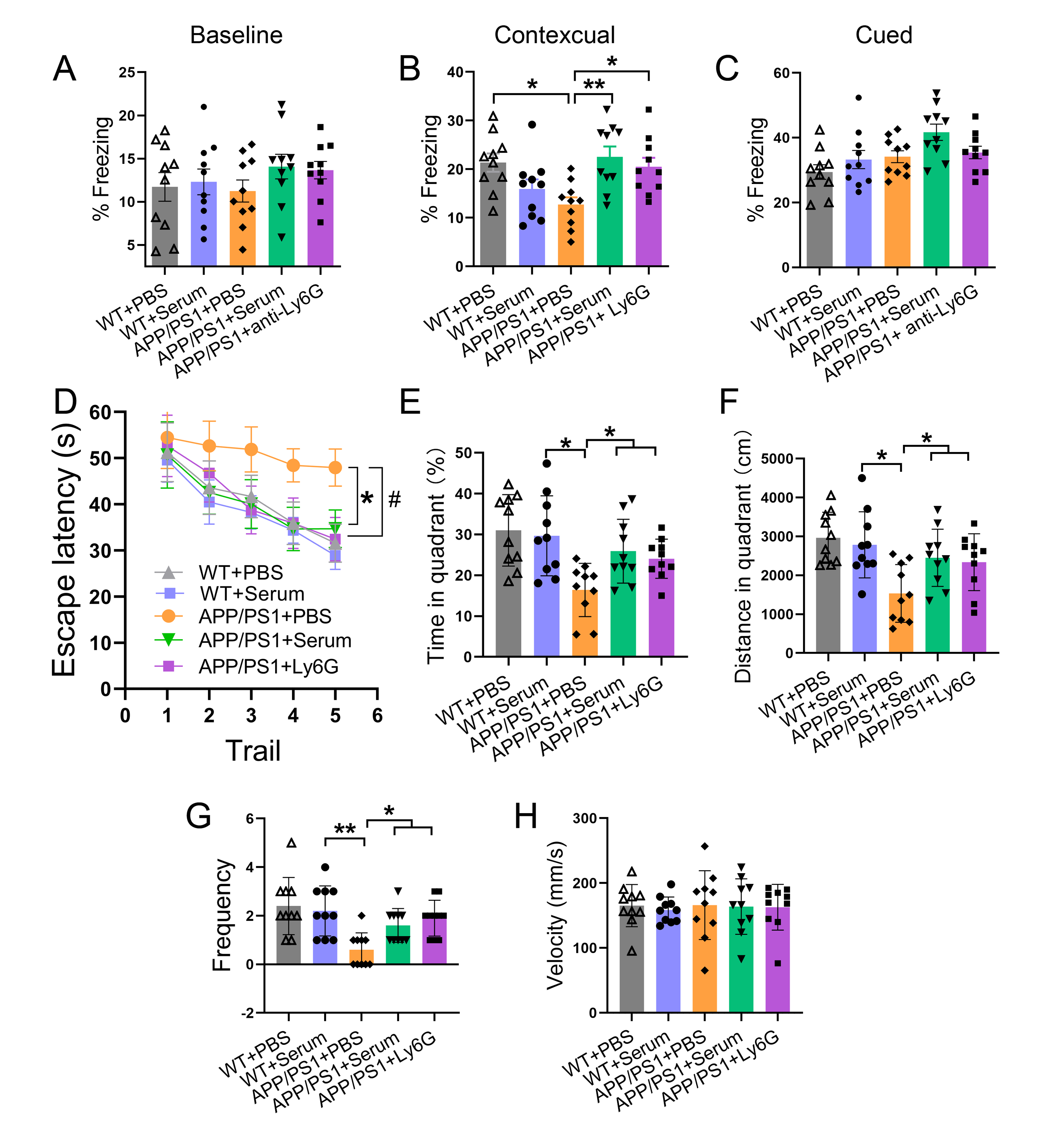

Supplement: Supplementary file 13 — Figure S9 [file 41380_2023_2097_MOESM13_ESM.tif]

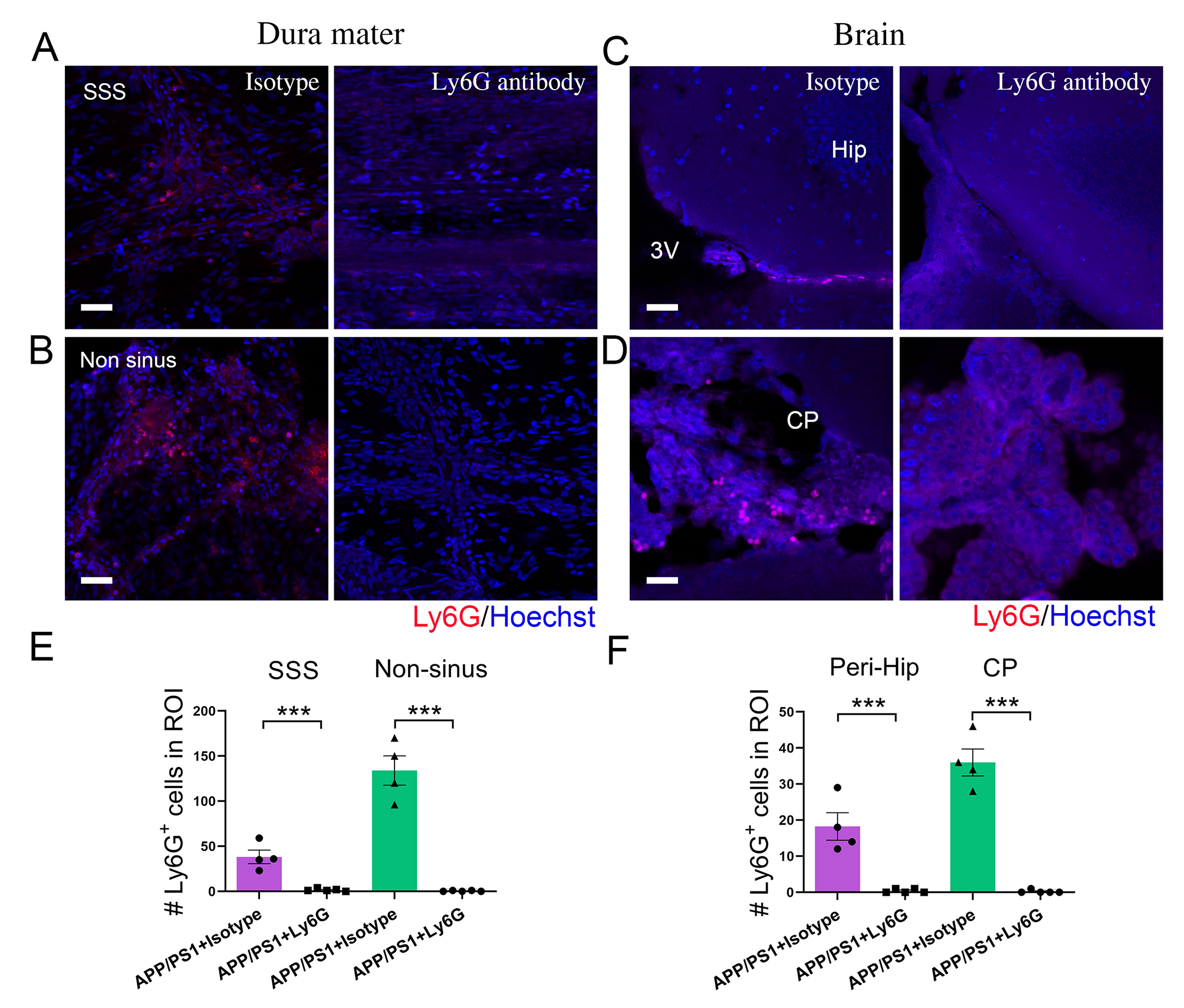

Supplement: Supplementary file 14 — Figure S10 [file 41380_2023_2097_MOESM14_ESM.tif]

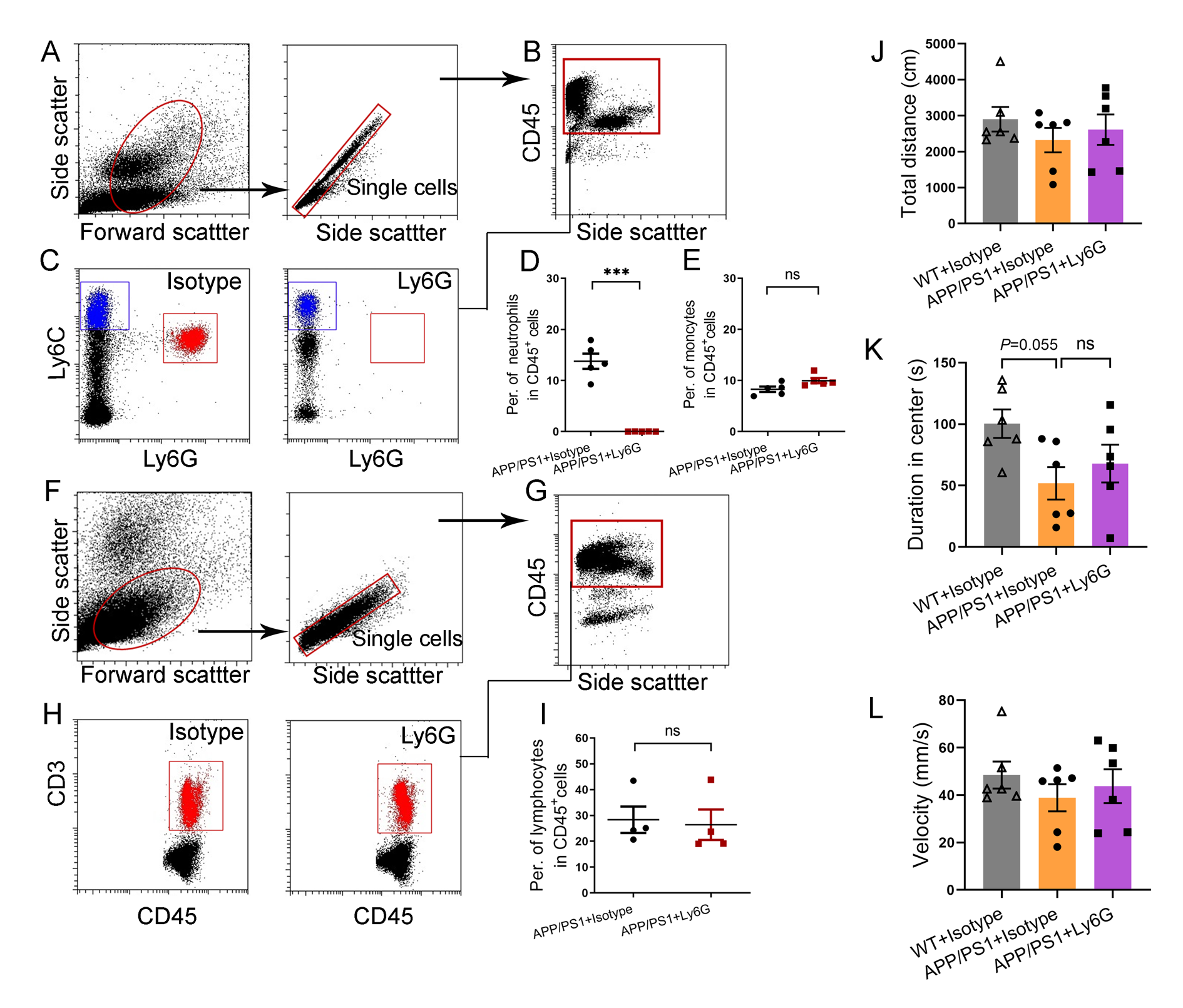

Supplement: Supplementary file 15 — Figure S11 [file 41380_2023_2097_MOESM15_ESM.tif]

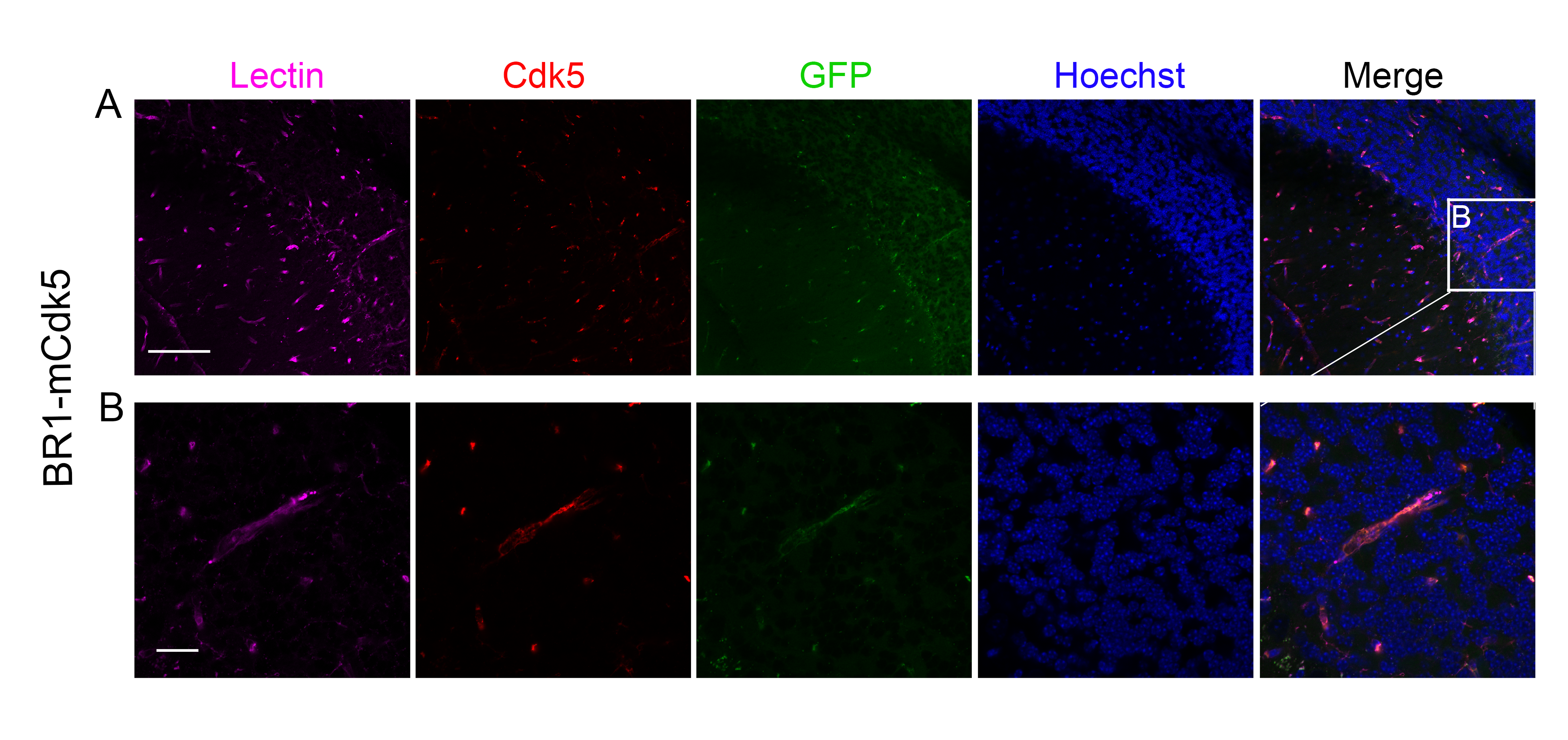

Supplement: Supplementary file 16 — Figure S12 [file 41380_2023_2097_MOESM16_ESM.tif]

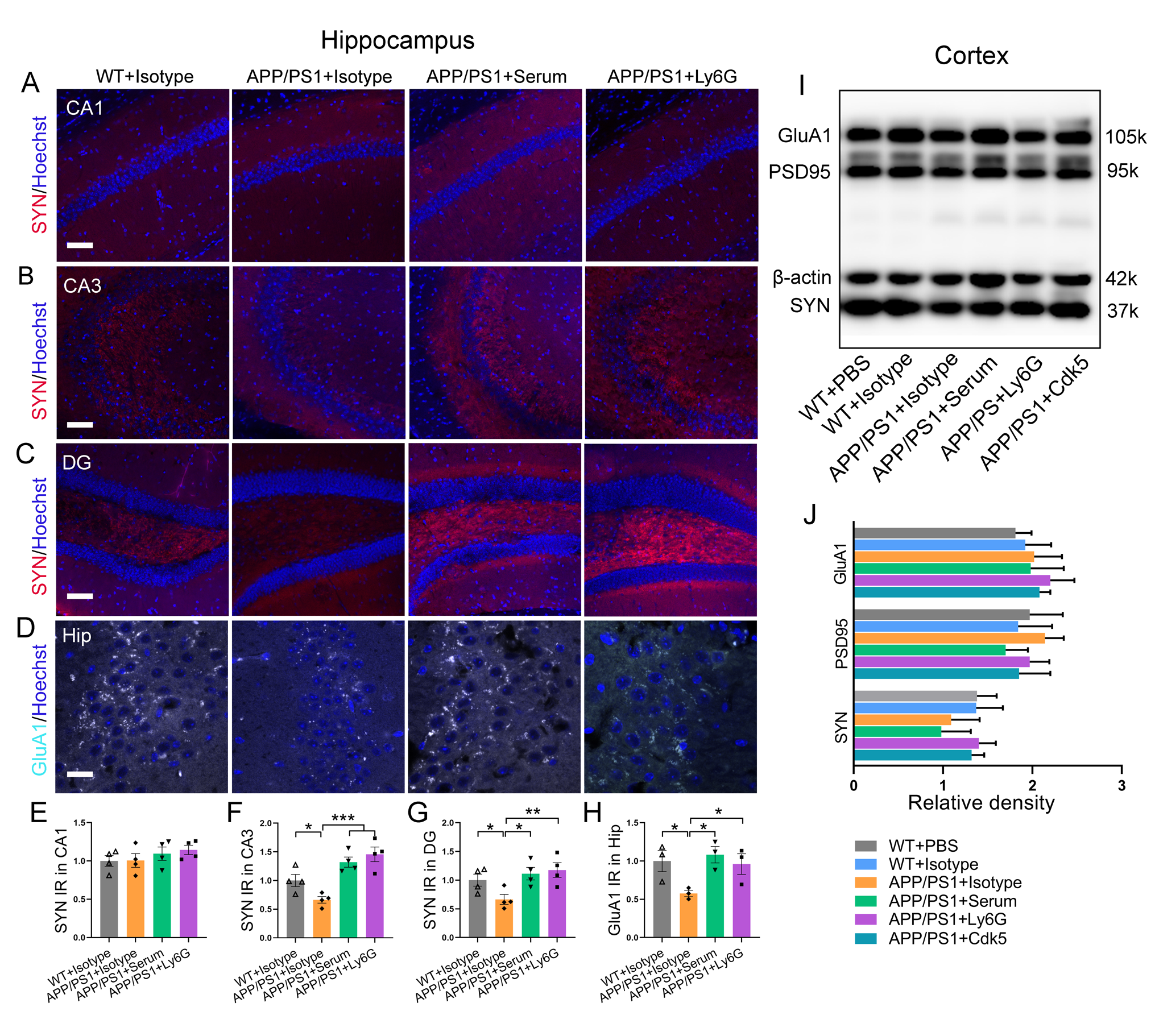

Supplement: Supplementary file 17 — Figure S13 [file 41380_2023_2097_MOESM17_ESM.tif]

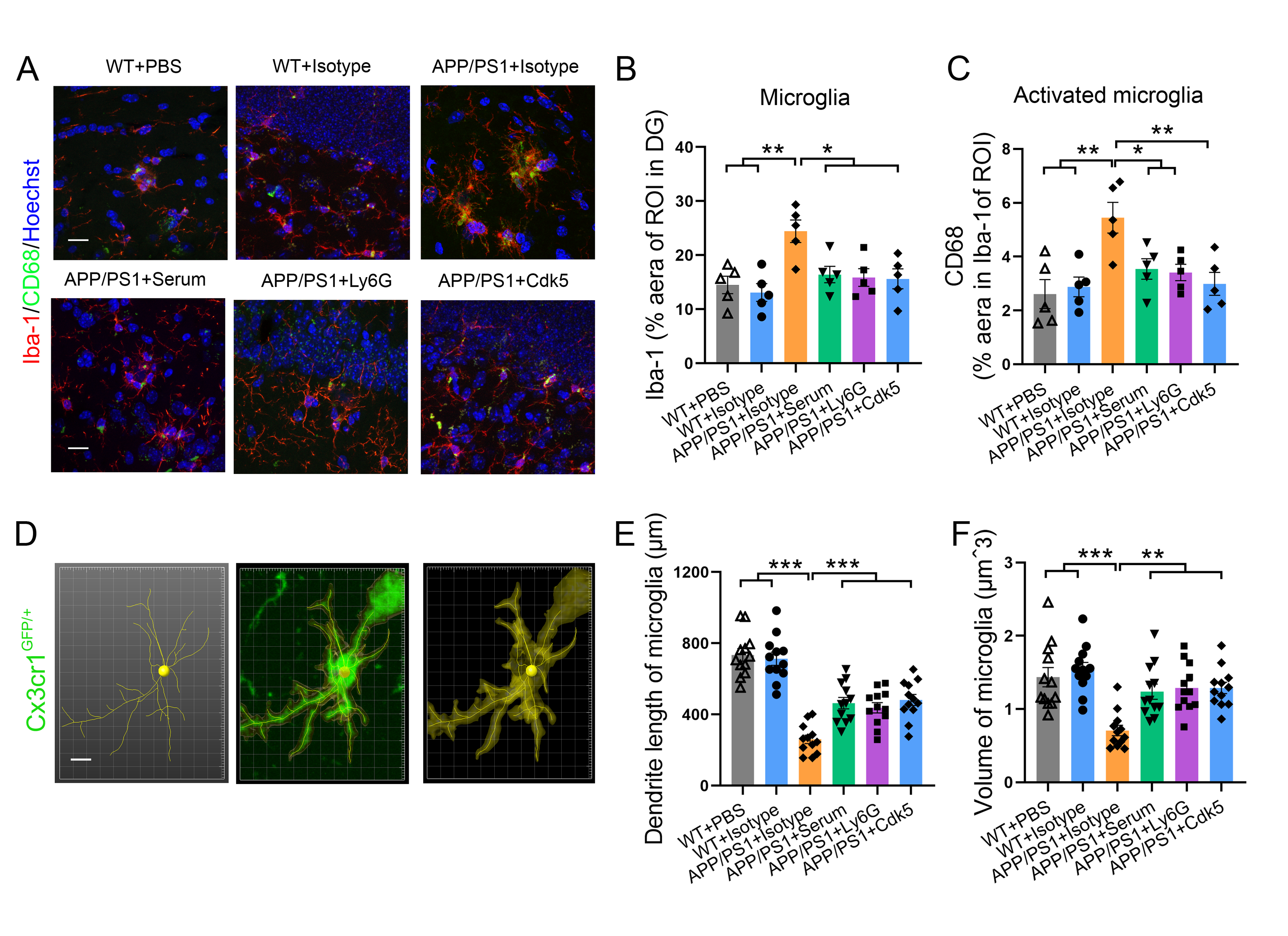

Supplement: Supplementary file 18 — Figure S14 [file 41380_2023_2097_MOESM18_ESM.tif]

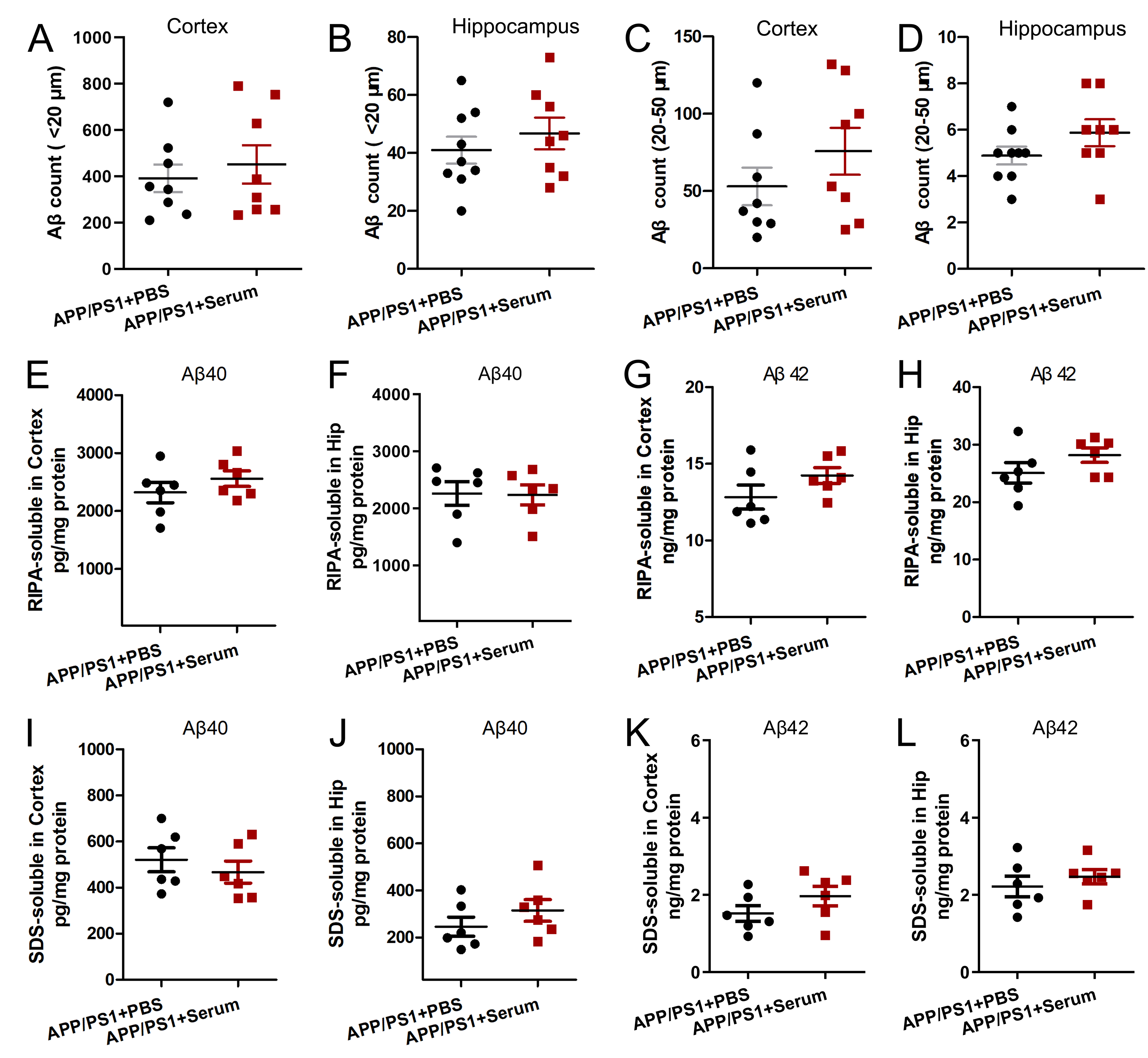

Supplement: Supplementary file 19 — Figure S15 [file 41380_2023_2097_MOESM19_ESM.tif]

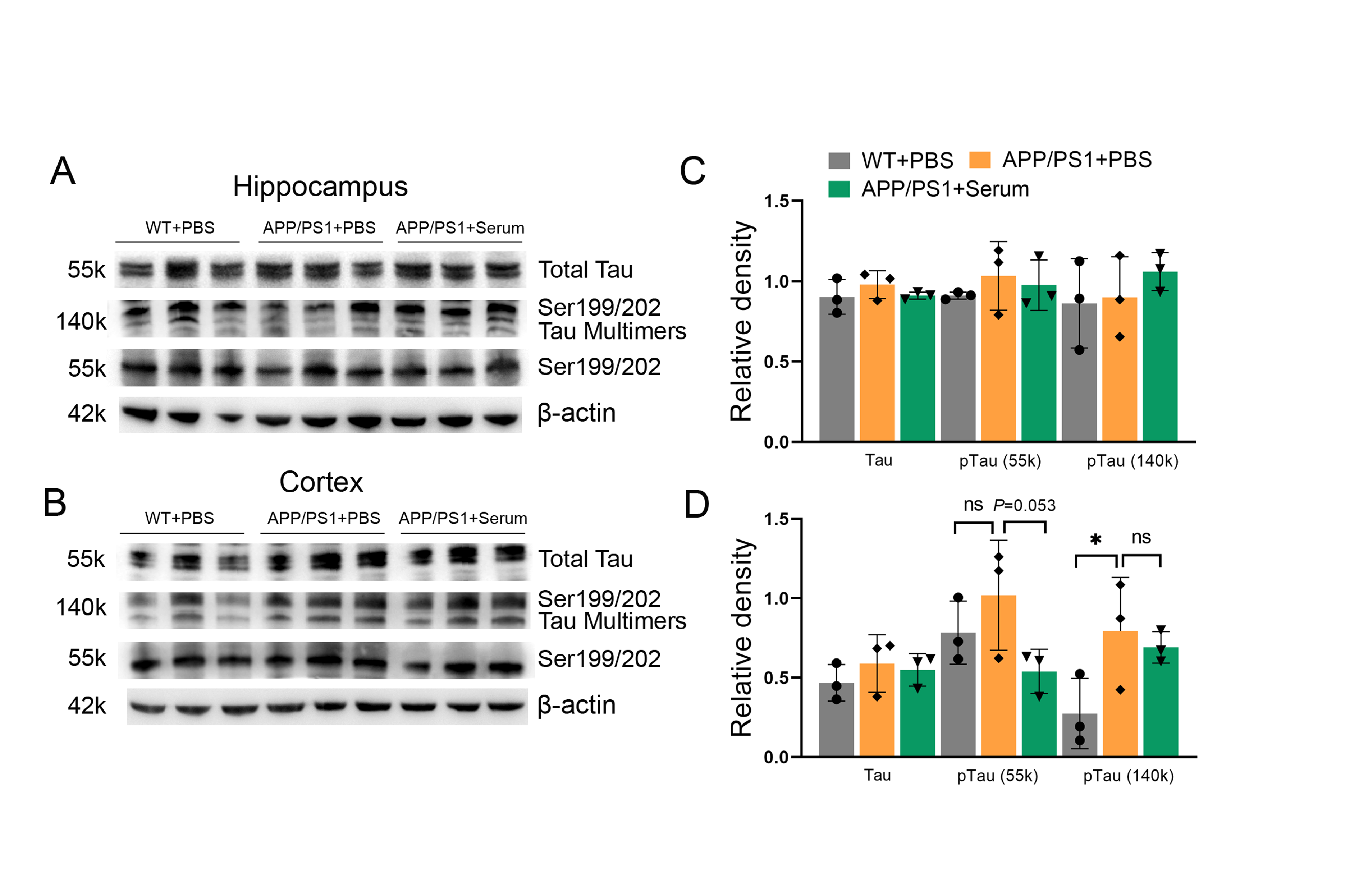

Supplement: Supplementary file 20 — Figure S16 [file 41380_2023_2097_MOESM20_ESM.tif]
